# Microbial Conversion of Glycerol Into 1,3‐Propanediol by Fermentation: Review of Fundamentals and Operational Strategies

**DOI:** 10.1111/1751-7915.70265

**Published:** 2025-11-14

**Authors:** María Fernanda Pérez‐Bernal, Roman Moscoviz, Xiaoli Wang, Nicolas Bernet, Eric Trably

**Affiliations:** ^1^ INRAE, Univ Montpellier, LBE Narbonne France; ^2^ SUEZ, Centre International de Recherche Sur l'Eau et l'Environnement (CIRSEE) Le Pecq France

**Keywords:** biomass immobilisation, bioprocess, metabolic pathways, mixed cultures, oxidation reduction potential

## Abstract

Over the past decades, biodiesel production has sharply increased worldwide and has led to an overproduction of glycerol, as by‐product. Therefore, glycerol is not only produced at low cost with a wide availability but is also a versatile precursor of useful value‐added chemicals such as1,3‐propanediol. At an industrial scale, glycerol conversion into 1,3‐propanediol is almost entirely carried out by fermentation processes as they have shown the best economic and environmental performances. The aim of this article is to provide an up‐to‐date state of the art on the fundamentals and fermentation process strategies for the microbial conversion of glycerol into 1,3‐propanediol. Glycerol fermentation metabolism is detailed and strategies concerning microbial inoculum (i.e., pure cultures of natural or genetically modified strains vs. mixed cultures or artificial consortia), process configuration (i.e., batch, fed‐batch and continuous reactors, biomass immobilisation) and related operational parameters (i.e., temperature, pH, oxido‐reduction potential) are discussed for the optimisation of 1,3‐propanediol production by fermentation.

## Graphical abstract

This review highlights recent advances in microbial fermentation strategies for converting glycerol, a biodiesel by‐product, into 1,3‐propanediol. It discusses metabolic pathways, microbial inocula and key operational parameters to optimise production, supporting sustainable chemical synthesis aligned with circular economy principles.

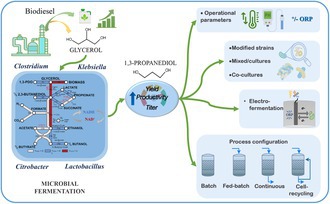

**Abbreviations**
1,3‐PDO: 1,3‐propanediol
ATP/ADP: adenosine tri/diphosphate
CAGR: compound annual growth rate
GRAS: generally recognised as safe
MFC: microbial fuel cell
NADH/NAD^+^
: nicotinamide adenine dinucleotide reduced/oxidised
OLR: organic loading rate
ORP: oxidation–reduction potential
PTT: polytrimethylene terephthalate
SHE: standard hydrogen electrode
Y_NADH_
: NADH yield (mol mol^−1^)

## 1 Introduction

Over the past decade, many countries, including the member states of the European Union and the United States, have adopted policies in favour of renewable energies. To reduce dependence on petroleum and limit their environmental footprint, these countries have favoured the development of alternative fuels for transportation. As a result, the global biofuel production has boomed since the 2000s with a substantial increase in biodiesel production. The global market is expected to reach USD 73.05 billion by 2030 (Research and Markets 2022). Most biodiesel is currently produced by transesterification of triglycerides issued from edible and non‐edible sources such as rapeseed oil, soybean oil, animal fat and microalgae (Akram et al. 2022). In this reaction, about 100 kg of glycerol is produced per ton of biodiesel (Marchetti et al. 2007; Ayoub and Abdullah 2012; Kosamia et al. 2020). Therefore, crude glycerol production has increased exponentially and could even reach up to 6.3 × 10^6^ t in 2025, with over 60% of its production issued from the biodiesel industry (Attarbachi et al. 2023). The crude glycerol oversupply has driven the price to drop from about 0.45 in 2001 to about 0.20 US $/kg in 2020 (Ciriminna et al. 2014; Attarbachi et al. 2023). Historically, glycerol was ranked among the 12 highest value‐added biomass‐derived chemicals for producing building block molecules such as 1,3‐propanediol (1,3‐PDO) (Werpy and Petersen 2004). Although no update ranking exists, the sustained growth and industrial success of 1,3‐PDO over the past two decades (Marr 2024) highlight glycerol's potential as a versatile feedstock for high‐value chemicals.

1,3‐PDO is particularly valuable for its role in producing a wide range of commodities, most notably polytrimethylene terephthalate (PTT) and polyurethane (PU) (da Silva Ruy et al. 2021), as well as adhesives, solvents and food‐related products. In 2015, 1,3‐PDO was traded on the US market at a price of 1.76 US $/kg (E4tech 2015). Currently, the global 1,3‐PDO market is estimated at USD 425.3 million and is projected to reach USD 813.1 million by 2032, corresponding to a compound annual growth rate (CAGR) of 9.7% (Coherent Market Insights 2025).

The first processes developed for 1,3‐PDO production were based on chemical transformation using acrolein or ethylene oxide (petroleum derivatives) as substrate (Kraus 2008; Lee et al. 2015). However, industrial production of 1,3‐PDO is fully supported by fermentation processes which offer economic advantages (e.g., operation at physiological temperature and atmospheric pressure) and environmental benefits (e.g., less energy consumption, lower greenhouse gas emissions) compared with petroleum‐based processes (Urban and Bakshi 2009; Erickson et al. 2012; van Heerden et al. 2023; Nimbalkar and Dharne 2024). Microbial1,3‐PDO production not only uses glycerol but also glucose. Indeed, the main substrate used in current large‐scale industrial production is glucose, which competes with food resources and raises sustainability concerns.

In this context, the development of environmentally friendly crude glycerol‐base 1,3‐PDO production remains an attractive yet underexploited opportunity for industry, despite the abundance and low cost of this biodiesel co‐product.

To address this gap, it is essential to integrate advances in glycerol fermentation metabolism with optimised inoculum, process configuration and operational strategies that can unlock its full potential for sustainable 1,3‐PDO production. Accordingly, this review aims to provide a comprehensive and up‐to‐date state of the art on the fundamentals and current process strategies for microbial fermentation of glycerol into 1,3‐propanediol. First, glycerol fermentation metabolism is presented, followed by strategies concerning the choice of an appropriate inoculum, process configuration and operational parameters and concluding with perspectives for the optimisation of 1,3‐PDO production by glycerol fermentation.

## 2 Fermentative Metabolism of Glycerol

**FIGURE 1:**
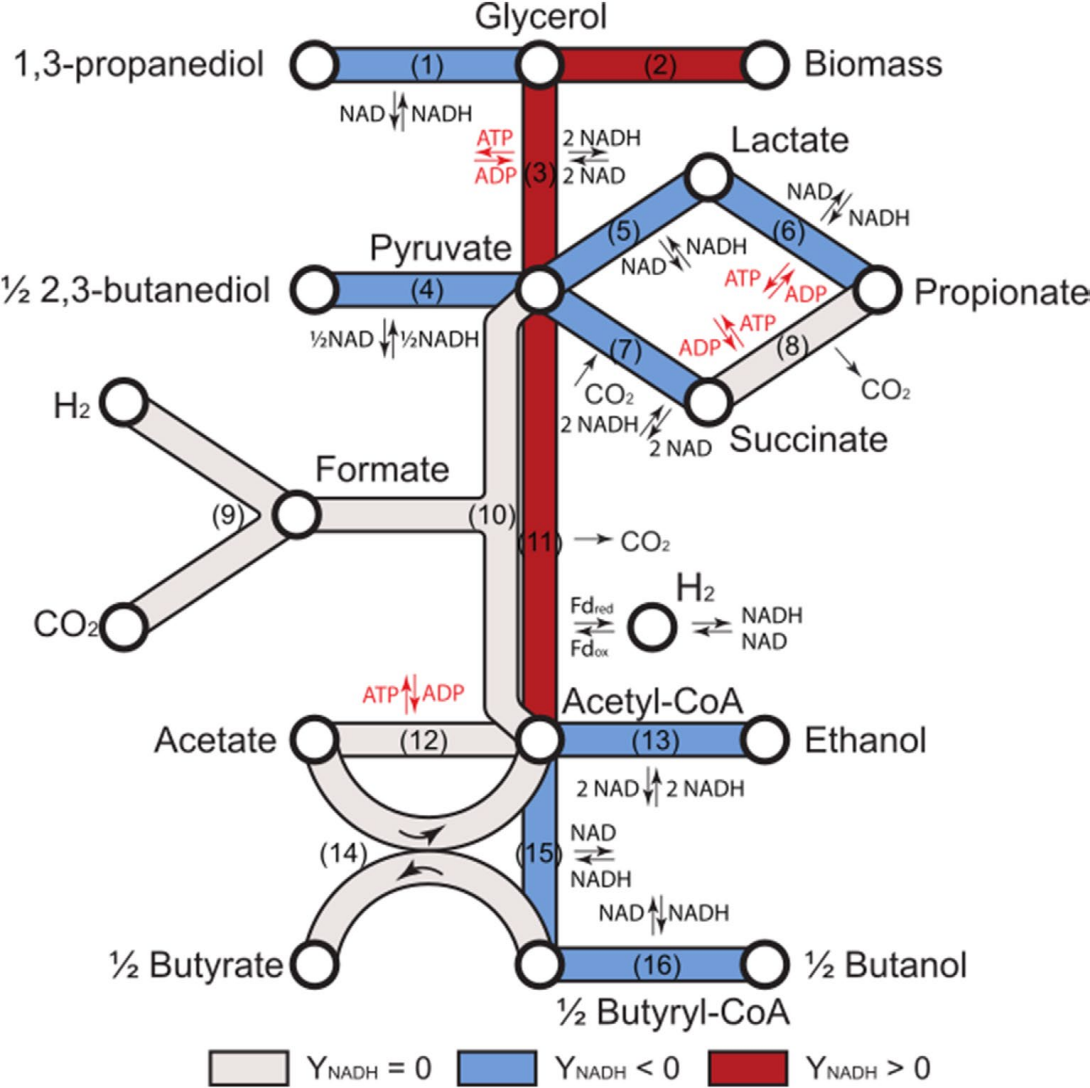
Simplified metabolic pathways of glycerol fermentation. Fd_ox_ and Fd_red_ stand for the oxidised and reduced form of ferredoxin, respectively. Numbers displayed on each pathway refer to reactions provided in Table 1. Adapted from Moscoviz, Toledo‐Alarcón, et al. (2016).

Understanding glycerol fermentation pathways is essential to optimising 1,3‐PDO production. Glycerol fermentation pathways have been extensively studied in model microorganisms such as *Klebsiella pneumoniae* (Zeng et al. 1993; Wang et al. 2003) or *Clostridium butyricum* (Zeng 1996). For both microorganisms, similar pathways have been reported and are summarised in Figure 1 and Table 1. Glycerol enters the cell, either by diffusion or active transport (Murarka et al. 2008; da Silva et al. 2009) to be used for both cell anabolism and catabolism. During anabolism, excess reducing equivalents are generated, because glycerol is, on average, more reduced than cell material (see Equation 2). Concerning catabolism during glycerol fermentation, it proceeds via two complementary branches: (i) an oxidative branch (i.e., Y_NADH_ ≥ 0), in which glycerol is oxidised to dihydroxyacetone and subsequently to pyruvate (glycolysis), while generating one mole of ATP and two moles of NADH per mole glycerol (see Equation 3), and (ii) a reductive branch (i.e., with an NADH yield, Y_NADH_ < 0), which dissipates NADH either through hydrogen evolution or through 1,3‐PDO formation (Equation 1).

**TABLE 1:** Condensed metabolic pathways of glycerol fermentation.

Reaction	Equation	References
Glycerol + NADH → 1,3‐PDO + H_2_O	(1)	Zeng et al. (1993), Zeng (1996)
Glycerol + ¾ NH_3_ + 7.5 ATP + 6 H_2_O → ¾ C_4_H_7_O_2_N + NADH	(2)	Zeng et al. (1993)
Glycerol → Pyruvate + ATP + H_2_O + 2 NADH	(3)	Zeng et al. (1993), Zeng (1996)
Pyruvate + ½ NADH → ½ 2,3‐Butanediol + CO_2_	(4)	Zeng et al. (1993), Ji et al. (2011)
Pyruvate + NADH → Lactate	(5)	Zeng et al. (1993), Zeng (1996), Temudo et al. (2007)
Lactate + NADH → Propionate + ATP + 2 H_2_O	(6)	Tholozan et al. (1992), Temudo et al. (2007)
Pyruvate + CO_2_ + 2 NADH → Succinate +2 H_2_O	(7)	Zeng et al. (1993), Temudo et al. (2007)
Succinate → Propionate + ATP + CO_2_	(8)	Schink et al. (1987), Temudo et al. (2007)
Formate + H_2_O → H_2_ + HCO_3_^−^	(9)	Zeng et al. (1993), Temudo et al. (2007)
Pyruvate + CoA + H_2_O → Acetyl‐CoA + Formate	(10)	Zeng et al. (1993), Temudo et al. (2007)
Pyruvate + CoA + H_2_O → Acetyl‐CoA + CO_2_ + Fd_red_	(11)	Zeng (1996), Temudo et al. (2007)
Acetyl‐CoA → Acetate + ATP + CoA + H_2_O	(12)	Zeng et al. (1993), Zeng (1996), Temudo et al. (2007)
Acetyl‐CoA + 2 NADH → Ethanol + CoA	(13)	Zeng et al. (1993), Zeng (1996), Temudo et al. (2007)
Butyryl‐CoA + Acetate → Butyrate + Acetyl‐CoA	(14)	Temudo et al. (2007), Louis and Flint (2009), Vital et al. (2014)
2 Acetyl‐CoA + 2 NADH → Butyryl‐CoA + CoA + H_2_O	(15)	Temudo et al. (2007), Louis and Flint (2009), Vital et al. (2014)
Butyryl‐CoA + 2 NADH → Butanol	(16)	Atsumi et al. (2008), Jin et al. (2011)

*Note:* For more readability, NAD^+^, H^+^ and ADP are omitted in the presented equations.

The partitioning of carbon through competing pyruvate‐derived pathways determines the availability of reducing equivalents for 1,3‐PDO synthesis and thus limits the overall yield. Under optimal conditions, when acetate is the sole by‐product, the highest theoretical yield reaches 0.72 mol_1,3‐PDO_ mol^−1^_glycerol_ (Moscoviz, Trably, et al. 2016). From pyruvate, carbon can be directed towards several well‐known fermentation end products. A first possibility is the conversion to lactic acid by the lactate dehydrogenase (Equation 5) which consumes NADH (Temudo et al. 2007). Lactate can subsequently be converted into propionate via the acrylate pathway with ATP generation (Equation 6) (Tholozan et al. 1992). Alternatively, pyruvate can be carboxylated to succinate (Equation 7) (Temudo et al. 2007) and further converted to propionate with the release of ATP and CO_2_ (Equation 8) (Schink et al. 1987). Some *Klebsiella*, *Enterobacter* and *Bacillus* species can also divert pyruvate to 2,3‐butanediol, which is however a chemical of industrial interest (Equation 4) (Ji et al. 2011).

In addition to these direct pyruvate conversions, carbon can also be funnelled through the conversion into the central intermediate acetyl‐coenzyme‐A (acetyl‐CoA), via either the pyruvate formate lyase (Zeng et al. 1993; Temudo et al. 2007) or pyruvate ferredoxin oxidoreductase pathways (Equations 10 and 11). During the pyruvate formate lyase pathway, formate can accumulate as a fermentation product, but can also be converted into carbonate and H_2_ by the formate‐hydrogen lyase complex (Equation 9) (Hallenbeck 2013; McDowall et al. 2014). On the other hand, the reduced ferredoxin produced during the pyruvate ferredoxin oxidoreductase pathway can contribute to electron dissipation through the production of H_2_ but can also be converted to NADH for further use of these electrons in other pathways (Hallenbeck 2013).

Acetyl‐CoA is a precursor of many fermentation end products. It can be converted into acetate with concomitant ATP production (Equation 12) (Zeng et al. 1993; Temudo et al. 2007), reduced to ethanol which dissipates NADH but does not release any ATP (Equation 13), or condensed into butyryl‐CoA and subsequently into butyrate or butanol (Equations 14–16).

Overall, all these oxidative pathways usually generate excess NADH that must be dissipated through reductive routes. In addition, NADH released during biomass synthesis also requires reoxidation to maintain redox balance during fermentation. For most microorganisms capable of glycerol fermentation, NADH dissipation occurs via two reductive pathways, which not yield directly to ATP and therefore do not contribute to anabolism. The first one is the 1,3‐PDO pathway (Zeng et al. 1993; Zeng 1996). It is important to note that the pathway leading to 1,3‐PDO differs between microbial groups, particularly in the initial reactions. In *Clostridium species*, glycerol is directly dehydrated to 3‐hydroxypropionaldehyde (3‐HPA) via a B_12_‐dependent glycerol dehydratase, which is subsequently reduced to 1,3‐PDO by 1,3‐propanediol dehydrogenase (Zeng 1996; Biebl et al. 1999). In contrast, *Enterobactariaceae* such as *Klebsiella* and *Citrobacter* can channel glycerol through two routes: (i) an oxidative route, in which glycerol is oxidised to dihydroxyacetone (DHA) and subsequently converted to dihydroxyacetone phosphate (DHAP) for glycolysis and energy generation, and (ii) a reductive route, in which glycerol is dehydrated to 3‐HPA and then reduced to 1,3‐PDO (Zeng et al. 1993; Mattam et al. 2013). In *Lactobacillus* species, the oxidative conversion of glycerol to DHA/DHAP predominates, while the reductive branch towards 1,3‐PDO serves primarily for redox balancing rather than for high‐yield production (Barbirato et al. 1998; da Silva et al. 2009). The second NADH dissipation route generates hydrogen from reducing equivalents, which depends on metabolic possibilities and, therefore, is strain dependent.

In summary, these competing pathways must be carefully considered when optimising 1,3‐PDO production, since they divert carbon and/or reducing equivalents away from the reductive branch. Among NADH‐dissipating routes, the 1,3‐PDO pathway is the most common dissipation pathway used by glycerol‐fermenting bacteria (Temudo, Poldermans, et al. 2008; Almeida et al. 2012; Clomburg and Gonzalez 2013). As a consequence, 1,3‐PDO is found most of the time as the end product, even though some species such as *Escherichia coli* have been reported to ferment glycerol exclusively into ethanol and H_2_ (Yazdani and Gonzalez 2007). Optimising 1,3‐PDO yield is thus nearly equivalent to maximising production of oxidised metabolites such as acetate.

## 3 1,3‐PDO‐Producing Bacteria

The microbial production of 1,3‐PDO has been reported for a variety of bacterial species capable of converting glycerol through the reductive pathway. Among these, members of the *Clostridiaceae*, *Enterobacteriaceae* and *Lactobacillus* genera are the most extensively studied due to their distinct physiological traits and metabolic capabilities. These bacterial groups differ in oxygen tolerance, cofactor requirements and biosafety level, which strongly influence their industrial applicability. To provide an overview, Table 2 summarises the general features of representative 1,3‐PDO‐producing species, including metabolic characteristics, co‐products and safety classification. Complementarily, Table 3 compiles reported production performances, titre, yield, productivity and glycerol source, allowing direct comparison of their efficiency under different fermentation conditions. Overall, *Clostridiaceae* strains typically exhibit the highest yields and versatility with crude glycerol, *Enterobacteriaceae* achieve high productivity under controlled conditions but often require vitamin B_12_ supplementation, and *Lactobacillus* species, although less efficient, offer GRAS status advantageous for food and cosmetic applications.

**TABLE 2:** General features of 1,3‐PDO‐producing bacteria.

1,3‐PDO producer	Oxygen tolerance	Sterile conditions	Biosafety level	Main by‐products	Best performance (highest 1,3‐PDO titre)	References
*Clostridiaceae*	−	+	1	Acetic acid Butyric acid	*C. butyricum* DL07 C_f_: 104.8 g L^−1^ Y: 0.65 mol_C_/mol_C_ Q: 3.38 g L^−1^ h^−1^	Wang et al. (2020)
*Enterobacteriaceae*	+	+	2	Acetic acid Lactic acid Ethanol 2.3‐butanediol	*K. pneumoniae* DSM 4799 C_f_: 80.2 g L^−1^ Y: 0.55 mol_C_/mol_C_ Q: 1.16 g L^−1^ h^−1^	Jun et al. (2010)
*Lactobacillus*	+	+	1 (+GRAS)	Acetic acid Lactic acid	*L. diolivorans* DSM 14421 C_f_: 85.4 g L^−1^ Y: 0.56 mol_C_/mol_C_ Q: 0.54 g L^−1^ h^−1^	Pflügl et al. (2012)
Engineered strains	+ or −	+	1–2	NA	Recombinant *E. coli* K‐12 C_f_: 135 g L^−1^ Y: 0.3 mol_C_/mol_C_ Q: 3.5 g L^−1^ h^−1^	Saxena et al. (2009)
Open mixed cultures	+	−	NA	Acetic acid Butyric acid Lactic acid Ethanol	Microbial consortium C2‐2M C_f_: 82.7 g L^−1^ Y: 0.66 mol_C_/mol_C_ Q: 3.06 g L^−1^ h^−1^	Zhou et al. (2017)

Abbreviations: C_f_, final 1,3‐PDO concentration; GRAS, generally recognised as safe by the Food and Drug Administration (FDA, USA); Q, 1,3‐PDO productivity; Y, 1,3‐PDO yield.

**TABLE 3:** Best 1,3‐PDO production performances reported in the literature.

Organism	Mode of operation	Titre (g L^−1^)	Yield (mol_C_ mol_C_^−1^)	Overall productivity (g L^−1^ h^−1^)	Glycerol source	References
*Clostridiaceae*						
*C. butyricum* CNCM1211	Batch	63.4	0.69	1.85	Crude	Barbirato et al. (1998)
*C. butyricum* AKR 102A	Fed‐batch	93.7	0.63	3.35	Refined	Wilkens et al. (2012)
*C. butyricum* IK124	Fed‐batch	87.0	0.65	1.90	Crude	Kaur et al. (2012)
*C. butyricum* DL07	Fed‐batch	104.8	0.65	3.38	Refined	Wang et al. (2020)
*C. butyricum* VPI 3266	Continuous	30.0	0.60	10.3	Refined	González‐Pajuelo, Andrade, and Vasconcelos (2005)
*C. butyricum* F2b	Continuous	35.0–48.0	0.67	2.90–5.50	Crude	Papanikolaou (2000)
*C. butyricum* DSM 5431	Continuous	26.6	0.63	13.30	Refined	Reimann et al. (1998)
*Enterobacteriaceae*						
*K. pneumoniae* ZJU 5205	Batch	63.1	0.65	5.74	Refined	Zhao et al. (2006)
*K. pneumoniae* DSM 4799	Fed‐batch	80.2	0.55	1.16	Crude	Jun et al. (2010)
*K. pneumoniae* LX3	Fed‐batch	68.2	0.62	3.43	Refined	Xue et al. (2010)
*C. freundii* FMCC‐B294	Fed‐batch	68.1	0.48	0.79	Crude	Metsoviti et al. (2013)
*K. pneumoniae* DSM 2026	Continuous	35.0–48.0	0.61	4.90–8.80	Refined	Menzel et al. (1997)
*Lactobacillus*						
*L. diolivorans* DSM 14421	Fed‐batch	92.0	0.51^a^	0.64	Refined	Lindlbauer et al. (2017)
*L. reuteri* ATCC 55730	Fed‐batch	65.3	0.19^a^	1.20	Refined	Jolly et al. (2014)
*L. reuteri* JH83 (mutant)	Fed‐batch	93.2	—^a^	1.29	Refined	Ju et al. (2021)
*Genetically engineered strains*						
*E. coli* K‐12 ER2925	Fed‐batch	104.4	—^b^	2.61	Refined	Tang et al. (2009)
*E. coli* K‐12	Fed‐batch	135.0	0.30	3.50	—^c^	Saxena et al. (2009)
*E. coli PK19‐DIQI*	Fed‐batch	80.0	0.99	1.67	Refined	Lee et al. (2025)
*K. pneumoniae* Cu ΔldhA	Fed‐batch	102.7	0.50	1.53	Refined	Oh et al. (2012)
*C. acetobutylicum* DG1 (pSPD5)	Fed‐batch	84.0	0.65	1.70	Refined	González‐Pajuelo, Meynial‐Salles, et al. (2005)
*Mixed cultures*						
Marine sludge	Batch	81.4	0.49	0.99	Refined	Jiang et al. (2017)
Microbial consortium C2‐2M	Fed‐batch	82.7	0.66	3.06	Crude	Zhou et al. (2017)
Biogas reactor sludge	Fed‐batch	70.0	0.56	2.60	Crude	Dietz and Zeng (2014)
*Co‐cultures*						
*A. faecalis* + *C. butyricum*	Batch	40.0	0.64	1.07	Crude	Szymanowska‐Powałowska et al. (2013)
*K*. sp. YT7 + *S. oneidensis* MR‐1	Fed‐batch	62.9	0.53	—	Refined	Wang et al. (2023)
*C. butyricum* + *E. coli/K. pneumoniae* (DUT)	Fed‐batch	77.7	0.62	—	Refined	Sun et al. (2022)

^a^ Fermentation with glucose as co‐substrate.

^b^ Fermentation was carried out with a glycerol/yeast extract mass ratio of 4. It was not possible to calculate an accurate carbon yield.

^c^ Glucose was used as a substrate.

### 3.1 Clostridiaceae

Many species from the *Clostridiaceae* family have been reported for their ability to convert glycerol into 1,3‐PDO (Saxena et al. 2009). Natural species that efficiently produce 1,3‐PDO are *Clostridium pasteurianum* (Dabrock et al. 1992; Biebl 2001), *Clostridium diolis* (Kubiak et al. 2012), *Clostridium bifermentans* (Kubiak et al. 2012), *Clostridium beijerinckii* (Wischral et al. 2016), *Clostridium perfringens* (Guo et al. 2017) and *Clostridium butyricum* (Papanikolaou 2000; Wang et al. 2020), the latter being the most studied. All these species produce 1,3‐PDO as the main metabolite during glycerol fermentation, along with a spectrum of co‐products depending on the type of species. For instance, *C. butyricum* generates mostly acetate, butyrate and lactate as co‐products, whereas *C. pasteurianum* produces acetate, butyrate, ethanol and butanol (da Silva et al. 2009; Kubiak et al. 2012). These species are strict anaerobes and spore‐forming, making them difficult to handle at an industrial scale (Jolly et al. 2014), but most 1,3‐PDO producers issued from this family are classified as biosafety level 1 (non‐pathogens) (U.S. Department of Health and Human Services 2009). In *C. butyricum* , the glycerol dehydratase, converting glycerol into 3‐hydroxypropionaldehyde (3‐HPA, intermediate for 1,3‐PDO production), is extremely sensitive to oxygen and is inactivated even at very low O_2_ levels (da Silva et al. 2009). As this enzyme is necessary in the reductive pathway of glycerol, its inactivation results in a complete stop of the fermentation process. It is also noteworthy to mention that this enzyme is vitamin B_12_‐independent in *C. butyricum* , unlike glycerol dehydratases in *Enterobacteriaceae* and *Lactobacillus* species (da Silva et al. 2009; Mattam et al. 2013; Liu et al. 2016). This implies that no supplementation of expensive vitamin B_12_ is required to sustain efficient production of 1,3‐PDO, resulting in lower operating costs. *C. butyricum* has been reported as one of the most efficient bacterial species for the production of 1,3‐PDO, from both refined and crude glycerol (Tables 2 and 3). A 1,3‐PDO concentration as high as 104.8 g L^−1^ with a yield of 0.65 mol_1,3‐PDO_ mol^−1^_glycerol_ and a productivity of 3.38 g L^−1^ h^−1^ were achieved in fed‐batch using *C. butyricum* DL07 fed with refined glycerol (Wang et al. 2020). A high 1,3‐PDO productivity of 10.3 g L^−1^ h^−1^ was also reached by using continuous systems with *C. butyricum* VPI 3266 fed with refined glycerol, with a final concentration of 30 g L^−1^ and a yield of 0.60 mol_1,3‐PDO_ mol^−1^_glycerol_ (González‐Pajuelo, Andrade, and Vasconcelos 2005).

### 3.2 Enterobacteriaceae

In contrast to the strictly anaerobic *Clostridiaceae*, many members of the *Enterobacteriaceae* family and more specifically from the *Klebsiella*, *Citrobacter* and *Enterobacter* genera able to ferment glycerol are easy to cultivate facultative anaerobes (Saxena et al. 2009; Jolly et al. 2014). However, most species are considered as classified as biosafety level 2 due to their opportunistic pathogenicity (U.S. Department of Health and Human Services 2009), which represents a significant constraint for their use at an industrial scale. The most efficient natural 1,3‐PDO producers from this family include *Enterobacter agglomerans* (Barbirato et al. 1995), *Klebsiella oxytoca* (Drozdzynska et al. 2011), *Citrobacter freundii* (Homann et al. 1990; Drozdzynska et al. 2014) and *K. pneumoniae* (Homann et al. 1990), the latter two being the most studied species. For all these species, 1,3‐PDO and acetate are the main products generated from glycerol fermentation, but secondary co‐products such as lactate, formate, succinate and ethanol can also be found depending on the type of strain and the culture conditions (da Silva et al. 2009). Within the *Enterobacteriaceae* family, *K. pneumoniae* is the species showing the best 1,3‐PDO production performances (see Table 2). Lower yields have been obtained with *K. pneumoniae* when compared to *C. butyricum* due to ethanol production, which dissipates NADH and outcompetes with 1,3‐PDO synthesis. Also, since the glycerol dehydratase of *K. pneumoniae* is vitamin B_12_‐dependent (Li et al. 2013; Mattam et al. 2013; Liu et al. 2016), yeast extract is often supplied in the fermentation medium increasing the overall cost of the process (Mattam et al. 2013). Nonetheless, a 1,3‐PDO concentration of 80.2 g L^−1^ was achieved using *K. pneumoniae* DSM 4799 in a fed‐batch fermenter fed with crude glycerol, with a productivity and a yield of 1.16 g L^−1^ h^−1^ and 0.55 mol_1,3‐PDO_ mol^−1^_glycerol_, respectively (Jun et al. 2010).

### 3.3 Lactobacillus

The *Lactobacillus* genus represents a distinct group of 1,3‐PDO producers characterised by their generally recognised as safe (GRAS) status (Pflügl et al. 2012; Kang et al. 2014) (biosafety level 1 (U.S. Department of Health and Human Services 2009)) and suitability for food and cosmetics applications (Jolly et al. 2014). Several species have been reported to produce 1,3‐PDO from glycerol (Saxena et al. 2009), such as *Lactobacillus brevis* (Schütz and Radler 1984), *Lactobacillus Buchner* (Schütz and Radler 1984), *Lactobacillus pains* (Kang et al. 2014), *Lactobacillus diolivorans* (Gottschal et al. 2002; Pflügl et al. 2012) and *Lactobacillus reuteri* (Jolly et al. 2014). Unlike *Clostridiaceae* and *Enterobacteriaceae*, none of these species can grow using glycerol as the sole carbon source, requiring co‐fermentation with sugars such as glucose. This limitation arises from the absence of key enzymes in the glycerol oxidative pathway (Schütz and Radler 1984; Gottschal et al. 2002; Jolly et al. 2014; Kang et al. 2014). For instance, *L. reuteri* lack the dihydroxyacetone kinase, an enzyme essential to connect glycerol to the glycolytic pathway. It is worth noticing that, similar to *Enterobacteriaceae* species, the glycerol dehydratase (the first enzyme of the glycerol reductive pathway) is vitamin B_12_‐dependent in *Lactobacillus* spp. (Schütz and Radler 1984; Pflügl et al. 2012; Jolly et al. 2014), resulting in a lower glycerol utilisation and 1,3‐PDO production when this vitamin is not sufficiently synthesised. When glucose and glycerol are used as co‐substrates, glycerol seems to be preferred for NADH dissipation through 1,3‐PDO production (Jolly et al. 2014). The glucose/glycerol ratio needs to be adjusted in order to maximise glycerol utilisation and 1,3‐PDO carbon yield. However, optimisation of this parameter seems species‐dependent, as increasing the glucose/glycerol ratio up to 1.5 resulted in an improvement of 1,3‐PDO productivity with *L. reuteri* (Jolly et al. 2014), while 1,3‐PDO production was nearly stopped when the ratio was over 0.3 with *L. diolivorans* (Pflügl et al. 2012). The best performance reached so far with natural *Lactobacillus* species was achieved by Lindlbauer et al. (2017) using *L. diolivorans* DSM 14421 in a fed‐batch fermenter supplied with a glucose/glycerol ratio of 0.1. A final 1,3‐PDO concentration of 92 g L^−1^ was attained, with a yield and a productivity of 0.51 mol_C‐1,3‐PDO_ mol^−1^_C‐substrate_ and 0.64 g L^−1^ h^−1^ respectively. Recently, this performance was surpassed by *L. reuteri* JH83 (Ju et al. 2021), a mutant strain obtained through electron beam irradiation mutagenesis to maintain its GRAS feature. This strain achieved a maximum 1,3‐PDO concentration of 93.2 g L^−1^ with a productivity of 1.29 g L^−1^ h^−1^ marking the highest 1,3‐PDO concentration reported for *Lactobacillus* species to date. A transcriptome analysis revealed changes in the expression levels of genes encoding sucrose phosphorylase, MFS transporter and muramyl ligase family proteins, which are associated with resistance to various stress factors, including high concentrations of organic acids.

## 4 Strategies to Improve Microbial 1,3‐Propanediol Production

### 4.1 Metabolic Engineering

**FIGURE 2:**
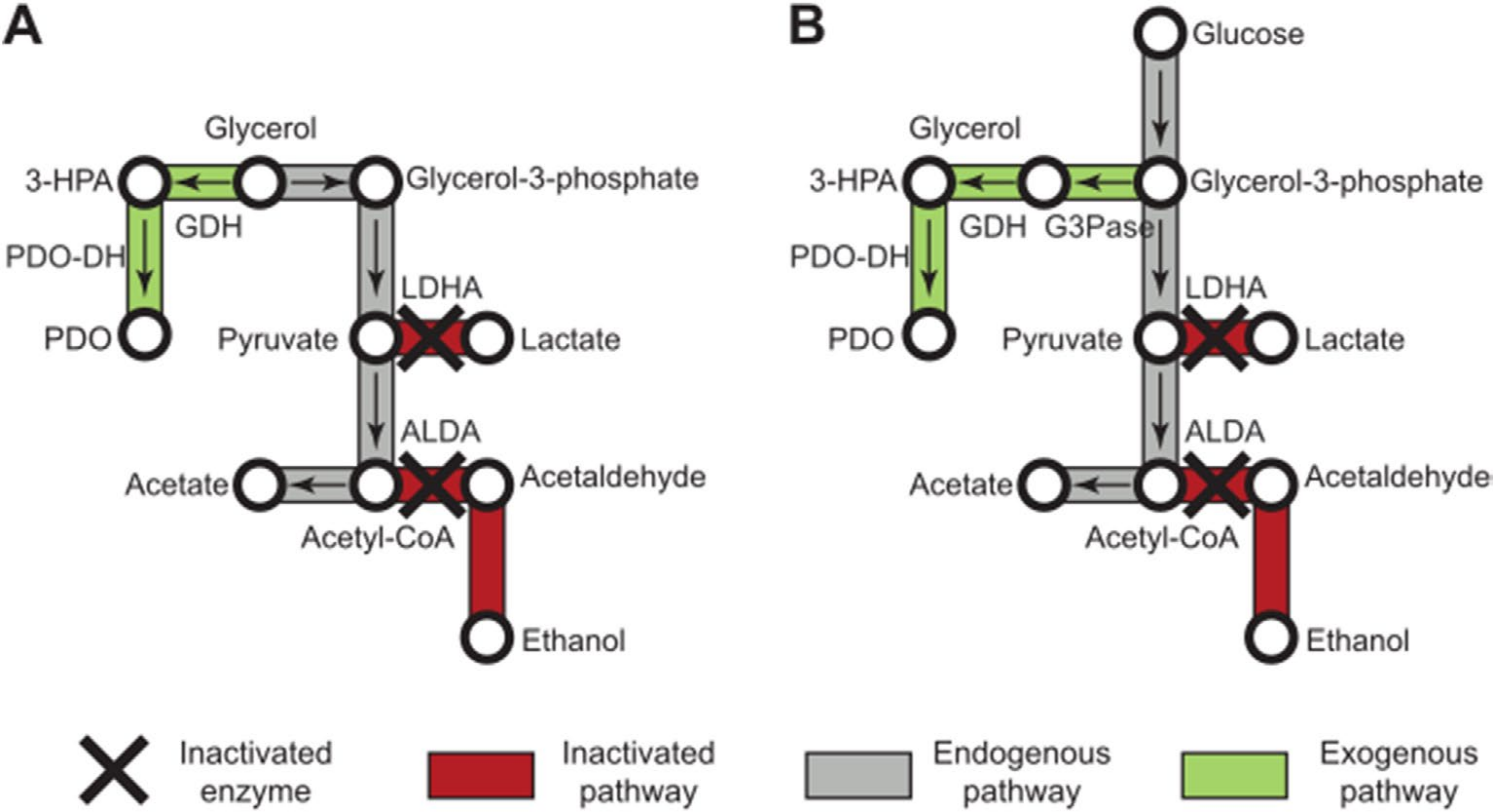
Genetic engineering strategies for (A) microorganisms lacking glycerol reductive pathway; (B) for producing 1,3‐PDO from glucose (simplified pathways). 1,3‐PDO‐DH, 1,3‐propanediol dehydrogenase; ALDA, aldehyde dehydrogenase; G3Pase, glycerol‐3‐phosphatase; GDH, glycerol dehydratase; LDHA, lactate dehydrogenase A.

Several strategies of genetic modifications have been investigated to improve the fermentative pathway of 1,3‐PDO. When looking at the glycerol oxidative metabolism, it is clear that 1,3‐PDO production is maximised during glycerol fermentation when acetate is the sole fermentation by‐product (see Section 2). Therefore, a first idea was to reduce the formation of co‐products such as lactate and ethanol. Zhang et al. (2006) inactivated the *aldA gene encoding the* acetaldehyde dehydrogenase in *K. pneumoniae* YMU2 (Zhang et al. 2006). As this enzyme is responsible for the conversion of acetyl‐CoA to acetaldehyde (precursor of ethanol), ethanol production was reduced by a factor of 5 when compared to the wild‐type strain. Simultaneously, 1,3‐PDO productivity and yield increased from 0.81 to 1.07 g L^−1^ h^−1^ and from 0.36 to 0.70 mol_1,3‐PDO_ mol^−1^_glycerol_, respectively. A similar strategy was conducted by Yang et al. (2006) to produce a lactate‐deficient strain by knocking out the *ldhA* gene encoding lactate dehydrogenase in *K. oxytoca* M5a1 (Yang et al. 2006). The 1,3‐PDO productivity and yield increased from 0.63 to 0.83 g L^−1^ h^−1^ and from 0.43 to 0.53 mol_1,3‐PDO_ mol^−1^_glycerol_, respectively, in regard to the wild‐type strain. Knocking out the same gene in *K. pneumoniae* Cu yielded one of the best 1,3‐PDO concentrations ever obtained. A final 1,3‐PDO concentration of 102.7 g L^−1^ was achieved in a fed‐batch fermentation using refined glycerol as substrate, with a productivity and a yield of 1.53 g L^−1^ h^−1^ and 0.50 mol_1,3‐PDO_ mol^−1^_glycerol_ respectively (Oh et al. 2012). Even though *C. butyricum* is one of the best natural 1,3‐PDO producers (see Table 3), genetic modifications of *C. butyricum* have been historically very challenging because of a lack of genetic engineering tools for this species (Kubiak et al. 2012; Fokum et al. 2021). Nonetheless, genes from *C. butyricum* encoding proteins involved in 1,3‐PDO synthesis have been successfully utilised in other microorganisms to improve its production. Indeed, a second method consists in adding genes that encode enzymes required for 1,3‐PDO synthesis in organisms lacking the glycerol reductive pathway (see Figure 2A). As such, a *C. acetobutylicum* recombinant was constructed by incorporating the pSPD5 plasmid containing the *dhaB1*, *dhaB2* and *dhaT* genes from *C. butyricum* , encoding for the B_12_‐independent glycerol dehydratase, its activating factor and the 1,3‐PDO dehydrogenase, respectively (González‐Pajuelo, Meynial‐Salles, et al. 2005). As a result, a high 1,3‐PDO concentration of 84.0 g L^−1^ was achieved while the wild‐type *C. acetobutylicum* DG1 was not able to produce 1,3‐PDO. In a similar way, the *E. coli* K12 strain was modified by incorporating the pBV220 plasmid containing the *dhaB1* and *dhaB2 genes from C. butyricum*, *and the yqhD* gene encoding 1,3‐PDO dehydrogenase from *E. coli* (Tang et al. 2009). The *dhaT*‐based 1,3‐PDO dehydrogenase can utilise solely NADH, while *yqhD*‐based 1,3‐PDO dehydrogenase can utilise both NADH and NADPH (Ma et al. 2010). Therefore, exploiting the *yqhD* gene instead of the *dhaT* gene from *C. butyricum* enables greater provision of reducing equivalents for 1,3‐PDO production. The recombinant mutant was able to produce up to 104.4 g L^−1^ of 1,3‐PDO with a productivity of 2.61 g L^−1^ h^−1^ in fed‐batch using refined glycerol (purity > 95%).

Another strategy is to connect glucose metabolism to the glycerol reductive pathway to produce 1,3‐PDO from glucose as alternative feedstock (see Figure 2B) (Nakamura and Whited 2003; Zong et al. 2017). A recombinant *K. pneumoniae* expressing the gene that encodes the glycerol‐3‐phosphatase of *Saccharomyces cerevisiae* was constructed by DuPont company (Saxena et al. 2009). This enzyme converts the glycerol‐3‐phosphate issued from glycolysis, into glycerol that can be further transformed into 1,3‐PDO. Using a similar approach in *E. coli* , the same company reported a 1,3‐PDO concentration of 135 g/L with a yield of 0.6 mol_1,3‐PDO_/mol_Glucose_ (Saxena et al. 2009; Liu et al. 2023). Recently, Lee et al. (2025) explored a novel strategy to optimise 1,3‐PDO production in *E. coli* K12 by coupling NADPH regeneration with targeted biosynthesis. In this approach, glucose was used exclusively for energy and glycerol for 1,3‐PDO synthesis. Glycolytic flux was redirected to the pentose phosphate (PP) and Entner–Doudoroff (ED) pathways to enhance NADPH production by deleting the *pgi* gene, which encodes glucose‐6‐phosphate isomerase. Additionally, genes responsible for the production of lactate, ethanol and acetate (*ldhA*, *adhE*, *pta‐ackA* and *poxB*) were deleted. Among the several strains constructed, *E. coli* PK19‐D1Q1 strain demonstrated the best performance in fed‐batch fermentation, achieving a titre, yield and productivity of 80.02 g L^−1^ of 1,3‐PDO, 0.99 mol_1,3‐PDO_ mol^−1^_glycerol_ and 1.67 g L^−1^ h^−1^, respectively. However, the use of glucose as a substrate or co‐substrate for producing commodity chemicals like 1,3‐PDO competes with food production and raises societal debates (Bardhan et al. 2015; Bharathiraja et al. 2016).

Recent advances in metabolic engineering such as the application of the CRISPR/Cas technology may offer more precise genetic modification opportunities and new perspectives to improve the yield of valuable compounds such as 1,3‐PDO (Lee et al. 2018; Fokum et al. 2019).

### 4.2 Open Mixed Microbial Cultures

Different studies have reported mixed cultures as an interesting alternative to pure culture for 1,3‐PDO production (Selembo et al. 2009; B. Liu et al. 2013; Dietz and Zeng 2014; Gallardo et al. 2014; Kanjilal et al. 2015; Xafenias et al. 2015; Moscoviz, Trably, et al. 2016; Moscoviz, Trably, and Bernet 2017; Zhou et al. 2017; Jiang et al. 2017; Varrone et al. 2017; Dahiya and Mohan 2021; Paranhos and Silva 2018). An open mixed culture consists of a mixture of several different bacteria that are maintained in a reactor often running under non‐sterile conditions. They are usually derived from natural inocula with a high initial microbial diversity (Temudo, Muyzer, et al. 2008). Drozdzynska et al. (2014) succeeded in isolating bacteria able to produce 1,3‐PDO from diverse sources such as groceries, soils, silages, composts, stagnant waters, sludge from municipal wastewater treatment plants and biogas fermenters (Drozdzynska et al. 2014). Microbial inocula from all these sources were suitable to carry out mixed culture fermentations. As open mixed culture fermentation can be operated under unsterile conditions, their operational costs are drastically reduced when compared to pure culture fermentations. Moreover, open mixed culture fermentations present other benefits such as better substrate utilisation, in situ production of nutrients by symbiotic species (e.g., growth factors and vitamins), the possibility of inhibitor removal or less sensitivity and benefiting from all kinds of syntrophic interactions in the community (Bode 2006; Kleerebezem and van Loosdrecht 2007; Li et al. 2014; Ghosh et al. 2016). As an advantage, minimal cultivation medium that does not contain expensive additives such as yeast extract can be used in open mixed culture fermentations. However, as a main drawback, the control of mixed‐culture fermentation is a complicated task as interspecies interactions within a bacterial consortium are complex and difficult to predict and control. When a population is efficiently oriented towards an efficient production of a specific metabolite, there is also no simple way to ensure its stability over time, to store or restore a functionality without changing the population structure. As a result, mixed‐culture processes often lack product specificity and are considered as less reproducible.

An important issue of using anaerobic mixed cultures is the potential formation of methane from 1,3‐PDO or glycerol (Dietz and Zeng 2014). This can dramatically decrease the fermentation performances. It is noteworthy to mention that several strategies can be applied to avoid methane production during glycerol fermentation with open mixed cultures (Selembo et al. 2009; B. Liu et al. 2013; Dietz and Zeng 2014; Gallardo et al. 2014; Kanjilal et al. 2015). First, the inoculum can be heat‐treated in order to remove non‐spore‐forming methanogens and select spore‐forming fermentative bacteria, that is, *Clostridium* sp. (Oh et al. 2003; Mei et al. 2016). Second, fermentations can be conducted at low pH (Oh et al. 2003) or with high carboxylic concentrations (Chen et al. 2008). Fermentations can also be conducted in a continuous reactor running at sufficiently low hydraulic retention time to prevent slow‐growing methanogens from remaining in the reactor (Oh et al. 2003). At last, specific inhibitors such as 2‐bromoethanesulphonate or 2‐bromoethanosulphonic acid (BES) can be added to the fermentation broth but that can be used for research purposes only (Zinder et al. 1984; Dahiya and Mohan 2021). Moreover, as high potassium or sodium concentrations can be found in crude glycerol (Marchetti et al. 2007) (used as alkali catalyst during transesterification), these conditions are not favourable to methanogens (Chen et al. 2008). As all these techniques are compatible with 1,3‐PDO production from glycerol, methane production is not a major issue for future implementation of open mixed‐culture processes for 1,3‐PDO production at large scale.

Regarding reactor performances, and more specifically 1,3‐PDO yield, several authors reported performances with mixed cultures close to the best results obtained with pure cultures. Using organic soil as inoculum, B. Liu et al. (2013) observed a 1,3‐PDO yield of 0.65 mol_1,3‐PDO_ mol^−1^_glycerol_ in batch mode which was close to the maximum theoretical yield of 0.72 mol_1,3‐PDO_ mol^−1^_glycerol_, although only 7 g L^−1^ of refined glycerol was used as substrate (B. Liu et al. 2013). With a similar inoculum and in batch fermentation at 35 g L^−1^ of crude glycerol, Kanjilal et al. (2015) reached a final 1,3‐PDO concentration of 19.4 g L^−1^, with a 1,3‐PDO yield of 0.67 mol_1,3‐PDO_ mol^−1^_glycerol_ (Kanjilal et al. 2015). So far, only a few studies have focused on optimising the final 1,3‐PDO concentration in open mixed cultures. Nonetheless, Dahiya and Mohan (2021) used combined dual pretreatment strategies involving heat treatment and the addition of BES, along with vitamin B_12_ supplementation, to selectively enrich a mixed consortium while inhibiting methanogens. These strategies enhanced 1,3‐PDO production in fed‐batch reactors with 20 g L^−1^ glycerol, achieving a final 1,3‐PDO titre of 9.3 g L^−1^ and a yield of 0.64 mol_1,3‐PDO_ mol^−1^_glycerol_. One of the best performances was reported by Zhou et al. (2017) in a fed‐batch fermentation fed with crude glycerol, with a 1,3‐PDO concentration of 82.7 g L^−1^, a yield and a productivity of 0.66 mol_1,3‐PDO_ mol^−1^_glycerol_ and 3.06 g L^−1^ h^−1^, respectively (see Table 3) (Zhou et al. 2017). These results are particularly remarkable as they were obtained without any pretreatment of the crude glycerol or any addition of yeast extract (or equivalent).

### 4.3 Co‐Cultivation or Artificially Designed Consortia

Artificially designed co‐culture systems have also been extensively studied for the production of various bio‐based chemicals (Zhang et al. 2018; Mittermeier et al. 2023). A co‐culture involves the cultivation of two or a few specifically selected species with known functions of interest. This is the main difference with open mixed cultures or natural consortia, which contain many unspecified species with unknown functions, potentially leading to lower product specificity and more difficult control. A co‐culture system is expected to expand the substrate spectrum, decrease the stress due to co‐product accumulation and develop more economical fermentation conditions for 1,3‐PDO production, compared to pure culture or mono‐culture fermentations. In addition, native microorganisms can only produce 1,3‐PDO using glycerol as a substrate. However, with a co‐culture system, this could be achieved using cheaper substrates such as glucose, starch or even CO_2_. For instance, a co‐culture experiment was carried out using *Zygosacharomyces rouxii* JL2011 (a glucose fermenter) and *Klebsiella pneumoniae* S6 to produce 1,3‐PDO (Ma et al. 2012). In mono‐culture fermentation, *Z. rouxii* JL2011 achieved a maximum glycerol yield of 35.5% (w/w) at a glucose concentration of 200 g L^−1^. In the co‐culture assays, the highest 1,3‐PDO concentration (15.2 g L^−1^) was obtained when *K. pneumoniae* S6 was inoculated after 96 h of fermentation under controlled pH conditions. In the pursuit of carbon neutrality, 1,3‐PDO production was demonstrated to be possible from CO_2_. A co‐culture consisting of engineered cyanobacteria *Synechococcus elongatus* strain YW1 and *Klebsiella pneumoniae* sp. achieved the production of up to 10 g L^−1^ of glycerol from CO_2_ for a final 1,3‐PDO titre after glycerol fermentation of 4.65 g L^−1^ (Wang et al. 2015).

Another advantageous combination relies on using an acid‐consumer partner. The inevitable generation of co‐products during glycerol fermentation can suppress continuous microbial metabolism and complicate the downstream purification process. Thus, a co‐culture of *Alcaligenes faecalis* and *Clostridium butyricum* was employed to convert raw glycerol to 40 g L^−1^ 1,3‐PDO (Szymanowska‐Powałowska et al. 2013). The yield of 1,3‐PDO was 0.64 mol_1,3‐PDO_ mol^−1^_glycerol_ with a productivity of 1.07 g L^−1^. *A. faecalis* consumed almost all lactate and acetate produced by *C. butyricum* , with only a remaining butyrate concentration below 1 g L^−1^. In a modelling study, Bizukojc et al. (2010) proposed a co‐culture of *C. butyricum* and the methanogenic archaea, *Methanosarcina mazei* , to relieve by‐product inhibition and utilise the by‐products for energy production. Among the scenarios explored, the optimal 1,3‐PDO production was achieved when *C. butyricum* did not produce hydrogen, enhancing acetate scavenging. Additionally, when methanol (commonly found in raw glycerol) is present, *M. mazei* could consume over 70% of the acetate, increasing methane production by up to 130%.

The potential of electroactive microorganisms (EAMs), typically exoelectrogenic bacteria such as *Geobacter* spp. and *Shewanella* spp., has also been explored in co‐cultures to increase the 1,3‐PDO yields. These bacteria are able to exchange electrons and reduced molecules that can be further used by other microorganisms. This extra reducing power can eventually be used to produce more 1,3‐PDO, needed to rebalance the intracellular redox state. The association of *G. sulfurreducens* and *C. pasteurianum* has been shown to increase by 37% the 1,3‐PDO yield from glycerol, from 0.18 to 0.24 mol_1,3‐PDO_ mol^−1^_glycerol_ (Moscoviz, de Fouchécour, et al. 2017). In this way, the production cost of 1,3‐PDO would be significantly reduced. This improvement, primarily attributed to interspecies electron transfer, was later proposed to be mainly induced by cobamides‐like molecules (Berthomieu et al. 2022). Additionally, Pérez‐Bernal et al. (2024) observed that the metabolic shift still occurs in the presence of an alternative electron acceptor such as fumarate and is moreover promoted. Recently, a novel *Klebsiella‐Shewanella* co‐culture was reported to enhance 1,3‐PDO yield from glycerol (Wang et al. 2023). In this system, *S. oneidensis* MR‐1 consumed the lactate produced by *K*. sp. YT7 and provided electrons to *K*. sp. YT7. During batch fermentation, the concentration of 1,3‐PDO, reached 32.30 g L^−1^, representing a 185.84% improvement compared to the *Klebsiella* monoculture. In fed‐batch fermentation, the final 1,3‐PDO concentration reached 62.90 g L^−1^, with a 1,3‐PDO yield of 0.53 mol_1,3‐PDO_ mol^−1^_glycerol_. Finally, some 1,3‐PDO producers, such as *C. butyricum* and *C. pasteurianum* , are strictly anaerobes and require redox potentials lower than −300 mV. Creating a reduced environment implies to remove oxygen traces. Thus, co‐cultures consisting of an oxygen scavenger and a 1,3‐PDO producer have been tested. Co‐culturing *K. pneumoniae* DUT2 or *E. coli* DUT3, as facultative anaerobic microbes, with *C. butyricum* DUT1, created the required anaerobic environment for *C. butyricum* and such artificially designed co‐culture achieved a maximum 1,3‐PDO concentration of 77.68 g L^−1^ with a yield of 0.62 mol_1,3‐PDO_ mol^−1^_glycerol_ (Sun et al. 2022).

### 4.4 Operating Parameters

Operational parameters play a crucial role in optimising 1,3‐PDO production, independently of the selected inoculum or process configuration. Among the most influent environmental parameters reported are: the availability of trace elements, the nitrogen and vitamins contents, impurities present in the substrate, temperature, pH and the redox conditions (e.g., presence of oxygen or other electron acceptor) (Stanbury et al. 2016). Trace elements, nitrogen and vitamins will not be discussed in this section as they are usually provided by complex additives such as yeast extract or tryptone prior to glycerol fermentation experiments, with no special focus on them in the published studies (apart from vitamin B_12_). Moreover, the use of crude glycerol as a substrate introduces additional variability in fermentation performances. The composition of crude glycerol depends on the biodiesel feedstock, the type and concentration of catalyst, and the methanol‐to‐oil ratio used during transesterification. For example, base‐catalysed biodiesel processes produce crude glycerol rich in glycerol and methanol with minor salts and esters, whereas acid‐catalysed processes yield lower glycerol, higher methanol and significant sulphuric acid content (Kumar et al. 2019). These impurities, collectively referred to as MONG (matter organic non‐glycerol), can exert positive or negative effects depending on the microbial strain, its metabolism and the impurity concentration. Consequently, crude glycerol streams from different sources may differ substantially in fermentability, and careful selection or pretreatment of the substrate may be necessary to achieve optimal 1,3‐PDO production. Refined glycerol provides more consistent results but at a higher cost. For more information regarding the impact of crude glycerol impurities over 1,3‐PDO production, readers may refer to Samul et al. (2014).

#### 4.4.1 Temperature

The most efficient 1,3‐PDO‐producing bacteria identified so far are mesophilic bacteria which grow typically between 20°C and 45°C (Willey et al. 2008). Finding optimal environmental parameters for model organisms such as *C. butyricum* and *K. pneumoniae* has been the focus of numerous studies using statistical designs (e.g., Plackett‐Burman, Taguchi) or kinetic models. For both species, temperature was reported to have only a slight effect on 1,3‐PDO yield and final concentration, in a wide temperature range from 30°C to 39°C (Oh et al. 2008; Rossi et al. 2013; Zhu and Fang 2013; Zhu et al. 2016). Nonetheless, a temperature close to 37°C has positive effects on the growth kinetics and 1,3‐PDO productivity. Therefore, 37°C is the optimal temperature for both *C. butyricum* and *K. pneumoniae* growth and 1,3‐PDO productivity. No further significant effect of temperature on 1,3‐PDO final concentration and yield was observed as well for less‐studied species such as *K. oxytoca* and *L. pains* in a similar range of temperature (30°C–39°C) (Grahame et al. 2013; Wojtusik et al. 2015). For these two species, 37°C was also optimal for both the growth rate and 1,3‐PDO productivity. Overall, only two species able to produce 1,3‐PDO are known to be importantly affected by temperature: *C. freundii* , with an optimal production temperature of 30°C (Metsoviti et al. 2013; Drozdzynska et al. 2014), and *B. pumilus* FMI3, which has an optimal temperature of 25°C for 1,3‐PDO production but predominantly produces polyhydroxyalkanoates at 40°C (Pinyaphong and La‐up 2024). For all other mesophilic species, a temperature between 35°C and 37°C is commonly retained as optimal for 1,3‐PDO production (Biebl 1991; Drozdzynska et al. 2014; Vieira et al. 2015).

It is noteworthy to mention that 1,3‐PDO production is also possible in thermophilic conditions. Wittlich et al. (2001) reported that, among 60 thermophilic enrichment cultures, 16 were able to produce 1,3‐PDO from glycerol (Wittlich et al. 2001), with a maximum concentration of 6.4 g_1,3‐PDO_ L^−1^. Isolates belonging to the genus *Caloramator*, such as *Caloramator boliviensis* (Crespo et al. 2012) and *Caloramator viterbensis* (Seyfried et al. 2002), were able to produce 1,3‐PDO at an optimal temperature of 60°C. *C. viterbensis* was even able to produce 1,3‐PDO with acetate as the sole by‐product, reaching a 1,3‐PDO yield of 0.69 mol_1,3‐PDO_ mol^−1^_glycerol_, very close to the theoretical maximum of 0.72 mol_1,3‐PDO_ mol^−1^_glycerol_ (Seyfried et al. 2002). Sittijunda and Reungsang (2020) also reported 1,3‐PDO production at thermophilic conditions (55°C ± 4°C), with a mixed culture in an UASB reactor. The best results were obtained when they increased the OLR from 25 to 75 g_glycerol_ L^−1^ d^−1^ with a final maximum titre of 7.5 g L^−1^ and a 1,3‐PDO yield of 0.36 mol_1,3‐PDO_ mol^−1^_glycerol_. The main by‐products were hydrogen and ethanol. Although thermophilic bacteria could be an interesting alternative for 1,3‐PDO production, very few studies focused on their use in optimised glycerol fermentation processes and the concentrations achieved are far from those obtained at mesophilic conditions (Wittlich et al. 2001; Sittijunda and Reungsang 2020).

#### 4.4.2 pH

The pH is usually described as a critical parameter affecting fermentation, because the catalytic activity of enzymes and, subsequently, the metabolic activity of microorganisms are highly dependent on extracellular pH (Pirt 1975). In particular, highly acidic and alkaline environments (i.e., pH < 4 and pH > 10 respectively) can be extremely toxic to bacterial activity. Toxicity at low pH is often related to weak acid accumulation such as volatile fatty acids (i.e., acetic acid, propionic acid and butyric acid) and lactic acid that are produced in their dissociate form during fermentation. As the pKa values of these acids range from 4 to 5 (around 4.8 for volatile fatty acids and 3.9 for lactic acid), an extracellular pH value below 4 would drastically increase the concentration of the undissociated form of these acids. These undissociated acids can freely diffuse inside the cells and cause proton imbalance (Colin et al. 2001). To maintain an optimal intracellular pH, these extra protons have to be actively removed from the cell by proton pumps while consuming ATP. This mechanism is directly competing with bacterial growth that can even be totally inhibited. A similar mechanism was reported for ammonia (NH_3_) inhibition at high pH. Ammonium (NH_4_^+^) is a common source of nitrogen that can be found in most fermentation media (Chen et al. 2008). The concentration of ammonia, which is the undissociated form of ammonium, increases as pH becomes high (pKa = 9.2). Ammonia is also membrane‐permeable and can cause proton imbalance as described with weak acids (Chen et al. 2008). Except for specific extremophiles, the pH range compatible with bacterial growth is usually comprised between 4 and 10.

**FIGURE 3:**
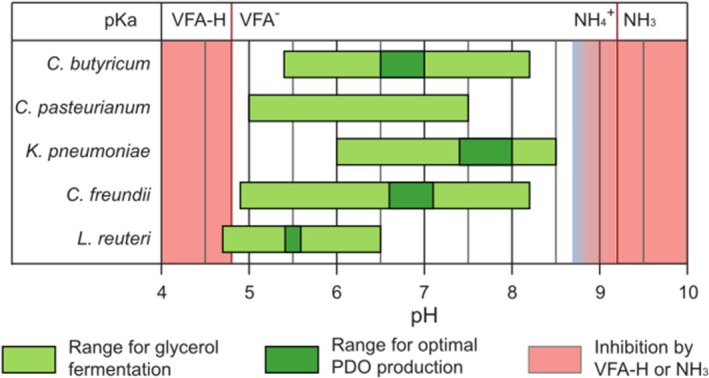
Diversity of pH ranges for glycerol fermentation and optimal 1,3‐PDO production for efficient 1,3‐PDO‐producing bacteria. VFA‐H/VFA^−^, undissociated/dissociated volatile fatty acids.

Concerning the effect of extracellular pH on glycerol fermentation, no single and common behaviour has been found for all 1,3‐PDO‐producing bacteria (see Figure 3). As for temperature, pH has been the focus of many optimisation studies, especially on *C. butyricum* and *K. pneumoniae*. Illustratively, *C. butyricum* is able to ferment glycerol at large pH values ranging between 5.4 and 8.2 (Zeng et al. 1994), with an optimal growth between pH 6.5 and 7.0 (Zeng et al. 1994; Zhu et al. 2016). *K. pneumoniae* is also capable of producing 1,3‐PDO from glycerol in a similar range of pH, that is, from 6 to 8.5 (Zhang et al. 2007; Sen et al. 2015) with an optimal value obtained in slightly alkaline conditions, that is, between 7.4 and 8 (Zhang et al. 2007; Oh et al. 2008; Hiremath et al. 2011). Interestingly, for *C. pasteurianum* , no clear dependence of the 1,3‐PDO production pathway on pH was observed for values ranging from 5.0 to 7.5 (Biebl 2001; Johnson and Rehmann 2016). *C. freundii* is able to grow on glycerol for a wide range of pH (4.6 to 8.2) with an optimal value for 1,3‐PDO production between 6.6 and 7.2 (Boenigk et al. 1993; Pflugmacher and Gottschalk 1994). Although no optimisation study of the pH was performed with *L. reuteri* , this species was reported to ferment glycerol at pH ranging between 4.7 and 6.5 and was more efficient for both 1,3‐PDO production and bacterial growth at pH 5.5 (Tobajas et al. 2009; Vieira et al. 2015). Finally, in mixed culture glycerol fermentation, strong functional redundancies within the microbial communities could ensure a robust and high 1,3‐PDO production for wide pH values ranging from 5 to 9 (Moscoviz, Trably, et al. 2016). Thus, the disparity of optimal range for 1,3‐PDO production observed with pure cultures can become an advantage concerning the use of mixed consortia for glycerol fermentation.

#### 4.4.3 Redox Potential (ORP)

The oxidation–reduction potential (ORP) of the fermentation medium, also called extracellular ORP, appears to be a relevant parameter to control the microbial metabolism (Wong et al. 2014; Zhu et al. 2014). Indeed, a fermentation process corresponds to a cascade of oxidation and reduction reactions that must be kept in balance. These reactions are mostly thermodynamically favourable and spontaneous, but they are also constrained by biological regulations within microorganisms and interspecies interactions in microbial communities. Similarly, to pH, which is a measure of the proton activity, the extracellular ORP corresponds to the activity of the electrons present in the medium. It is mainly affected by temperature, the chemical composition of the medium and the degree of reduction of the metabolites produced by fermentation. It can be easily measured with an ORP sensor located in the medium. The extracellular ORP is particularly important because it can subsequently affect the intracellular ORP (C.‐G. Liu et al. 2013). Intracellular ORP, representing the redox state inside a cell, can be estimated through the NADH/NAD^+^ ratio because of the intracellular redox homeostasis (C.‐G. Liu et al. 2013). Intracellular ORP is known to influence gene expression and enzyme synthesis, which can further cause shifts in the metabolic pathways and impact the whole metabolism (C.‐G. Liu et al. 2013; Harrington et al. 2015). Chemical control of the extracellular ORP by supplying chemical reductive or oxidative agents, or bubbling gases (Hallenbeck et al. 2012) has already been successfully implemented to improve the production of 1,3‐PDO (Du et al. 2006).

A first approach consists of providing oxygen (aeration or micro‐aeration) to facultative anaerobes, such as *Enterobacteriaceae* or *Lactobacillus* species, in order to enhance their growth and subsequently improve the 1,3‐PDO production rate. Oxygen is one of the electron acceptors having the highest potential. Indeed, microorganisms generate more energy (i.e., ATP) when using oxygen than under complete or partial anaerobiosis. Such ORP control through the O_2_‐addition strategy was successfully applied to *K. pneumoniae*, improving the productivities in batch fermentation from 1.62 to 2.94 g L^−1^ h^−1^ under anaerobic and micro‐aerobic conditions, respectively (Chen et al. 2003). This improvement correlated with a higher bacterial biomass growth but also anti‐correlated with 1,3‐PDO yield (from 0.57 to 0.52 mol_1,3‐PDO_ mol^−1^_glycerol_) as a result of the competition between O_2_ and 1,3‐PDO as NADH sink. For lactic acid bacteria such as *L. diolivorans* (Pflügl et al. 2012) and *L. reuteri* (Jolly et al. 2014), O_2_ supply under both micro‐aerobic and aerobic conditions successfully enhanced the biomass production and the kinetics. However, 1,3‐PDO productivities and yields were always lower in the presence of oxygen and 1,3‐PDO production was even completely stopped under aerobic conditions (i.e., pO_2_ = 0.20). Thus, micro‐aeration and aeration are efficient ways for accelerating biomass formation, but the 1,3‐PDO pathway can be outcompeted. In fact, two‐stage processes could be a way to take advantage of aeration: a first aerobic stage for increasing biomass production rate and a second anaerobic stage for producing 1,3‐PDO at higher yield and increased productivity (Du et al. 2006; Tang et al. 2009).

Another strategy consists of controlling the extracellular ORP by a loop system supplying both oxidising and reducing agents. As 1,3‐PDO production has the unique function of ensuring redox homeostasis in glycerol fermentation (Johnson and Rehmann 2016), its production is tightly related to the environmental redox conditions. Du et al. (2006) investigated the response of *K. pneumoniae* M5aL to a redox control environment at three levels (−140, −190 and −240 mV vs. SHE) (Du et al. 2006). Interestingly, ORP changes resulted in a significant redistribution of the metabolic fluxes: rising ORP from −240 to −140 mV versus SHE increased the acetate production by 2.5‐fold while decreasing lactate accumulation by threefold. 1,3‐PDO production was also affected by the extracellular ORP and an optimum for both its production and bacterial growth of this strain was found at −190 mV versus SHE. The same strain was then used in mutagenesis experiments to select mutants able to efficiently grow at low potentials (Du et al. 2007). A mutant having a preferred ORP for growth of −280 mV versus SHE was able to produce 60% more 1,3‐PDO (69.6 g_1,3‐PDO_ L^−1^) than the wild strain (42.5 g_1,3‐PDO_ L^−1^) in fed‐batch fermentation. The NADH/NAD^+^ ratio in this mutant was twice as high as than in the parent strain throughout the fermentation time. This could have contributed to enhance the activity of the 1,3‐PDO dehydrogenase and consequently accelerate the 1,3‐PDO production. Similarly, Zhu et al. (2014) observed that *K. oxytoca* shifted its metabolism when the extracellular ORP decreased from −150 to −240 mV versus SHE (Zhu et al. 2014). Lower ORP accelerated glycerol consumption and enhanced 1,3‐PDO production while reducing bacterial growth. Proteomic analysis revealed that the abundance of 1,3‐PDO dehydrogenase encoded by the *yqhD* gene increased by sevenfold when extracellular ORP decreased from −140 to −240 mV versus SHE. As 1,3‐PDO production was enhanced at lower potential, it is probable that this upregulation was coupled with a higher availability of NADH, as reported with *K. pneumoniae* by Du et al. (2007). Overall, all the reported studies have shown that extracellular ORP can influence the intracellular NADH/NAD^+^ ratio through metabolic regulations and subsequently redistribute the metabolic fluxes. For *K. pneumoniae* and *K. oxytoca* , low extracellular ORP was related to an enhancement of the 1,3‐PDO production as long as the strains were able to survive.

### 4.5 Process Configuration

Beyond environmental parameters, the choice of process configuration is a key determinant of 1,3‐PDO production performance. In addition to the environmental factors, each configuration involves operational parameters directly linked to the mode of operation, such as dilution rate and substrate concentration, which influence fermentation outcomes. Biotechnologies for 1,3‐PDO production are characterised by lower environmental impacts and possibly the lowest operating costs compared to chemical and petrochemical techniques. However, simple fermentation processes such as batch fermentation usually offer low reaction rates and relatively low product concentrations. As a consequence, huge volumes of fermentation broth must be treated to extract and purify the product. In the case of 1,3‐PDO, downstream processing for isolation and purification can account for up to two‐thirds of the overall production cost (Pandey et al. 2016). Therefore, optimising both the final product concentration and the purification/extraction procedures is a key element for making the biotechnological production of 1,3‐PDO cost‐competitive (E4tech 2015). Among the different factors influencing these outcomes, the chosen process configuration and cell handling strategy have a major impact on fermentation performance, as shown in Table 4, which compares reported performances of suspended versus immobilised or recirculated cells under batch, fed‐batch and continuous operation modes.

**TABLE 4:** Comparison between suspended and immobilised/recirculated culture fermentation performances.

Mode of operation		Best performance^a^				References
		Final titre g_1,3‐PDO_ L^−1^	Yield mol_C_/mol_C_	Productivity g L^−1^ h^−1^	Microorganism	
Batch	Suspended	81.4	0.49	0.99	Marine sludge	Jiang et al. (2017)
	Immobilised	63.1	0.65	5.74	*K. pneumoniae* ZJU 5205	Zhao et al. (2006)
Fed‐batch	Suspended	104.8	0.65	3.38	*C. butyricum* DL07	Wang et al. (2020)
	Immobilised^b^	86	0.63	4.2	*C. butyricum* SCUT343‐4	Lan et al. (2021)
Continuous	Suspended (w/o recirculation)	30	0.60	10.3	*C. butyricum* VPI 3266	González‐Pajuelo, Andrade, and Vasconcelos (2005)
	Immobilised/with recirculation	26.6	0.63	13.3	*C. butyricum* DSM 5431	Reimann et al. (1998)

^a^ Best performance reported corresponds to the highest 1,3‐PDO concentration reported for batch and fed‐batch fermentation. For continuous fermentation, it corresponds to the study showing the highest productivity with a final concentration > 20 g_1,3‐PDO_ L^1^.

^b^ Repeated fed‐batch with immobilised cells.

#### 4.5.1 Batch Mode

Batch fermentation is the simplest mode of reactor operation, where fermentation is carried out at constant volume, without any feed inlet or outlet sampling and is often an effective and economic solution for slow processes. As reported in Table 4, suspended cultures in batch mode can achieve competitive final concentrations; however, the corresponding productivities remain limited compared to other configurations. In order to achieve significant final 1,3‐PDO concentrations in batch fermentations, several authors attempted to use high initial glycerol concentrations. Unfortunately, glycerol concentrations ranging from 60 to 110 g L^−1^ were reported as inhibitory for both the bacterial growth and 1,3‐PDO production in the model microorganisms *C. butyricum* (Biebl 1991; Szymanowska‐Powałowska 2015; Zhu et al. 2016) and *K. pneumoniae* (Cheng et al. 2005). This inhibitory effect is related to a probable osmotic stress caused by such a high concentration of substrate (Kubiak et al. 2012). Therefore, several approaches have been implemented to select 1,3‐PDO producers with a high resistance to osmotic stress. For instance, a chemical mutagenesis approach was used to select resistant *C. diolis* strains (Otte et al. 2009). As a result, while the wild‐type strain DSM 15410 could tolerate a maximal glycerol concentration of 62 g L^−1^, a selected *C. diolis* mutant demonstrated a 77% increase in tolerance, reaching 109 g L^−1^. Even with the selective procedure, no single strain has been reported to support more than 110 g L^−1^ without observation of strong inhibitory effects. Nonetheless, a microbial consortium has recently been reported as able to tolerate refined glycerol concentrations up to 200 g L^−1^ while producing 81.4 g L^−1^ of 1,3‐PDO, at a yield and productivity of 0.63 mol_1,3‐PDO_ mol^−1^_glycerol_ and 0.99 g L^−1^ h^−1^ respectively (see Tables 3 and 4) (Jiang et al. 2017). This final 1,3‐PDO concentration is significantly higher than the highest titre of 63.4 g L^−1^ achieved with single natural strains in batch mode (Barbirato et al. 1998), thus indicating the great potential of bacterial consortia regarding future fermentation processes.

#### 4.5.2 Fed‐Batch Mode

In fed‐batch mode, the substrate is supplied to the bioreactor during fermentation and the products remain in the reactor until the end of the run (i.e., no outlet) (Yamanè and Shimizu 1984). Usually, the bioreactor is fed either continuously (e.g., constantly or exponentially) or intermittently in response to a control parameter (e.g., pH, pO_2_ or other on‐line measurements). This allows improved substrate utilisation. Since substrate concentrations can be maintained at low levels, the fed‐batch operation mode is an efficient way to limit substrate inhibition (Yamanè and Shimizu 1984; Tang et al. 2013). It is also an effective process to accumulate targeted end products and to achieve final concentrations higher than in batch mode, which is highly desirable regarding downstream purification processes (Kaur et al. 2012). However, the accumulation of fermentation products can induce several types of stress to micro‐organisms and eventually inhibit cell growth and product formation (Colin et al. 2001; Kubiak et al. 2012). Regarding glycerol fermentation, it was hypothesised that 1,3‐PDO accumulation could inhibit cell growth by modifying membrane organisation through an increase in the cell membrane fluidity (Colin et al. 2000; Huffer et al. 2011). Membrane ATPase and transport mechanisms could also be inhibited by 1,3‐PDO as reported for other alcohols (Colin et al. 2000; Huffer et al. 2011). The mechanisms of 1,3‐PDO inhibition were investigated for several strains of *C. butyricum* and *K. pneumoniae* (Biebl 1991; Zeng et al. 1994; Colin et al. 2000; Xue et al. 2010; Szymanowska‐Powałowska and Kubiak 2015). Similar results were reported for both species and 1,3‐PDO was found to be inhibitory of microbial growth in a range of 60–90 g L^−1^ for the wild‐type strains (Zeng et al. 1994; Colin et al. 2000; Szymanowska‐Powałowska and Kubiak 2015). Strong inhibition was also reported for by‐products such as acetic and butyric acids which were found to totally inhibit microbial growth at concentrations of 27 and 19 g L^−1^, respectively (sum of both dissociated and undissociated form) (Colin et al. 2000). Despite these limitations, Wang et al. (2020) reached a final 1,3‐PDO concentration of 104.8 g L^−1^ with a yield and productivity of 0.65 mol_1,3‐PDO_ mol^−1^_glycerol_ and 3.38 g L^−1^ h^−1^, respectively, using *C. butyricum* DL07 in a fed‐batch reactor fed with refined glycerol. This is the best performance achieved so far using natural 1,3‐PDO producers in fed‐batch fermentation mode (see Table 3). The use of engineered strains made it possible to overcome the limitations due to 1,3‐PDO inhibition by using strains more resistant to 1,3‐PDO than *C. butyricum* and *K. pneumoniae* . For instance, in a fed‐batch reactor using glucose as substrate and an *E. coli* K‐12 recombinant strain, Laffend et al. (2000) were able to reach a maximum 1,3‐PDO concentration of 135 g L^−1^ (Saxena et al. 2009). As a consequence, fed‐batch processes are those that are currently used for 1,3‐PDO production at an industrial scale because of their highest final concentrations and the resulting lowest downstream process costs (Kaur et al. 2012; Wang et al. 2020).

#### 4.5.3 Continuous Fermentation

Continuous operation has been explored as a way to sustain higher reaction rates and reduce downtime. Table 4 shows that continuous fermentations typically result in significantly higher productivities compared to batch and fed‐batch processes, although issues such as strain stability and contamination risk can limit long‐term operation. In fed‐batch fermentations, initial 1,3‐PDO productivity is high while it drops dramatically in the later period of fermentation due to product inhibition (Tang et al. 2013). Continuous fermentation is a way to set substrate and product concentrations at a constant level by continuously removing fermentation products while providing nutrients at the same rate (Westgate and Emery 1990). This mode of operation offers other advantages over batch and fed‐batch fermentations, such as precise control of microbial growth rate through dilution rate adjustment (Tang et al. 2013) and the possibility of reaching a steady state (i.e., variables become time independent), which are convenient for process control and further downstream purification processes (Maxon 1955). However, in such systems, final product concentrations are usually lower than those achieved in fed‐batch mode; thus, continuous fermentation represents a compromise between final concentration and productivity. Both *C. butyricum* and *K. pneumoniae* showed good performances for continuous 1,3‐PDO production. With dilution rates ranging between 0.1 and 0.25 h^−1^, *K. pneumoniae* DSM 2026 was able to produce between 35.2 and 48.5 g_1,3‐PDO_ L^−1^ at a yield and productivity of 0.61 mol_1,3‐PDO_ mol^−1^_glycerol_ and 4.9–8.9 g L^−1^ h^−1^, respectively (Menzel et al. 1997). This productivity was approximately 2–3.5‐fold higher than those obtained in batch and fed‐batch cultures with the same *K. pneumoniae* strain. The best 1,3‐PDO productivities achieved so far in classic continuous fermentation (i.e., no cell recycling or immobilisation) were observed with *C. butyricum* VPI 3266 at a dilution rate of 0.30 h^−1^. A concentration of 30 g_1,3‐PDO_ L^−1^ was attained with a yield and productivity of 0.60 mol_1,3‐PDO_ mol^−1^_glycerol_ and 10.3 g L^−1^ h^−1^, respectively (González‐Pajuelo, Andrade, and Vasconcelos 2005).

However, maintaining culture stability during extended continuous operation remains challenging, as strain degeneration, genetic drift or washout can occur over time, leading to reduced metabolic activity or altered profiles (Biebl et al. 1999). These issues are particularly critical for strict anaerobes such as *C. butyricum* , which may lose native plasmids under prolonged selective pressure. To overcome such limitations, several strategies have been developed to retain high and active biomass within the reactor, including cell recycling and immobilisation, which are discussed in the next section.

#### 4.5.4 Cell Recycling and Immobilisation

Several strategies have been implemented to increase the cell density in glycerol fermentation. Indeed, high cell concentrations offer several advantages. These include increased fermentation kinetics (Gungormusler‐Yilmaz et al. 2015) and improved tolerance to high concentrations of glycerol and other fermentation co‐products (Reimann et al. 1998; Gungormusler‐Yilmaz et al. 2015). To some extent, achieving high cell concentration could be a solution to overcome substrate and product inhibitions, as observed in classic suspended fermentation processes. Additionally, increasing cell density can also improve the process productivity.

One approach to achieve high cell concentrations consists of recycling the cells in continuous systems by passing the culture through a permeable membrane. The cell‐free liquid can then be used for downstream purification process while the concentrated cell suspension is reinjected into the reactor (Reimann et al. 1998; Avci et al. 2014). The best performances using this approach were reached by Reimann et al. (1998) using *C. butyricum* DSM 5431 at a dilution rate of 0.5 h^−1^, with a final concentration, yield and productivity of 26.6 g_1,3‐PDO_ L^−1^, 0.63 mol_1,3‐PDO_ mol^−1^_glycerol_ and 13.3 g L^−1^ h^−1^, respectively. This productivity was about 30% higher than the best results obtained in classic continuous fermentation (10.3 g L^−1^ h^−1^). However, in general, membrane clogging is a frequent issue in cell recycling systems, making it difficult to maintain high and stable performances over time. This limitation hinders the industrial‐scale implementation of cell recycling.

Another approach to increase cell concentration is immobilising the microbial cells inside the reactor (Gungormusler‐Yilmaz et al. 2015). Several techniques have been explored, such as cell aggregation (e.g., self‐immobilisation as granules), cell attachment (e.g., biofilm formation on inert support) or cell entrapment (e.g., in porous materials). These techniques are applied in continuous systems (e.g., packed‐bed reactor, fluidised‐bed reactor) as well as in repeated‐batch and fed‐batch processes. For instance, encapsulating *K. pneumoniae* ZJU 5205 in a repeated‐batch process, a final concentration, yield and productivity of 63.1 g_1,3‐PDO_ L^−1^, 0.65 mol_1,3‐PDO_ mol^−1^_glycerol_ and 5.74 g L^−1^ h^−1^, respectively, were achieved by Zhao et al. (2006). While the final concentration obtained in this study was lower than the best batch fermentation performance achieved with suspended cultures (see Table 4), the productivity was improved nearly sixfold. Similar improvements were reported for continuous fermentation using immobilised cultures. For example, immobilising *K. pneumoniae* on ceramics balls, increased 1,3‐PDO productivity from 4.9 g L^−1^ h^−1^ (suspended cultures) to 9.8 g L^−1^ h^−1^ (Gungormusler et al. 2011b). A comparable 2.5‐fold productivity increase was reported for cultures of *Clostridium beijerinckii* immobilised on pumice stones (Gungormusler et al. 2011a). Cell immobilisation on calcium alginate beads has also been applied to *Bacillus pumilus* FMI3, a strain resistant to inorganic salts. After optimisation, batch fermentation with crude glycerol produced a maximum titre of 44.1 g_1,3‐PDO_ L^−1^ and a yield of 0.89 mol_1,3‐PDO_ mol^−1^_glycerol_, with acetate as the only by‐product. This approach improved PDO yield 2.86‐fold compared to non‐immobilised cells (Pinyaphong and La‐up 2024). Additionally, *Clostridium butyricum* SCUT343‐4 immobilised in a fibrous‐bed bioreactor demonstrated high performance in a repeated‐batch fermentation. After seven cycles, the process achieved a 1,3‐PDO titre of 86 g L^−^1, a yield of 0.63 mol_1,3‐PDO_ mol^−1^_glycerol_, and a productivity of 4.20 g L^−1^ h^−1^, representing a 2.36‐fold productivity improvement compared to batch fermentation (Lan et al. 2021).

One of the main disadvantages of these systems is the typically lower product titres which may render downstream purification steps costlier. This trade‐off between productivity and final concentration must be carefully optimised to ensure overall process competitiveness (Zeng and Biebl 2002). In practice, an adequate balance between reactor throughput and product concentration is essential to minimise purification costs. Therefore, while cell immobilisation effectively enhances reactor performance and process stability, their industrial application requires a balanced optimisation of cell density, productivity and product concentration. Although limited studies have focused on cell immobilisation for 1,3‐PDO production, they all show that the choice of optimal immobilisation material is largely strain‐dependent (Gungormusler‐Yilmaz et al. 2015). Therefore, no unique and optimal support material for 1,3‐PDO fermentation processes has yet been reported.

### 4.6 Electro‐Fermentation

Another method used to direct the metabolic flux in glycerol fermentation towards 1,3‐PDO production is the use of electrodes in bio‐electrochemical reactors in a process known as electro‐fermentation (Moscoviz, Toledo‐Alarcón, et al. 2016; Virdis et al. 2022). The presence of electrodes in the bulk offers the opportunity to provide or remove electrons from the system at the cathode or the anode, used as electron donor or acceptor, respectively, thus influencing the electron balance of the fermentation process or indirectly the ORP conditions. So far, only a few studies have focused on glycerol electro‐fermentation for 1,3‐PDO production (Zhou et al. 2013, 2015; Choi et al. 2014; Xafenias et al. 2015; Moscoviz, Trably, and Bernet 2017; Harrington et al. 2015; Utesch et al. 2019). They are all based on the same basic concept: as 1,3‐PDO production from glycerol is an electron dissipation pathway, providing extra electrons to microorganisms with a cathode working at low potential should be beneficial to the process. Some of the studies even set the cathodic potential to produce H_2_ electrochemically (Zhou et al. 2013, 2015; Xafenias et al. 2015). Zhou et al. (2013) improved the 1,3‐PDO production yield from 0.25 to 0.50 mol_1,3‐PDO_ mol^−1^_glycerol_ by applying a potential of −900 mV versus SHE at a cathode in mixed culture fermentation (Zhou et al. 2013). Another study showed that the presence of a cathode poised at a relatively high potential of 45 mV versus SHE (i.e., without electrochemical H_2_ production) shifted the fermentation pattern in *C. pasteurianum* towards more 1,3‐PDO production with very low energy consumption (Choi et al. 2014). This result suggests that this microorganism was able to uptake electrons directly from the cathode (i.e., without redox mediators such as H_2_ or formate) and that the electron consumption had a higher effect on *C. pasteurianum* metabolism than just dissipating electrons (e.g., reduced biomass production, complete change of fermentation patterns). Recently, a novel strain of *Pseudomonas aeruginosa* (EL14) was isolated from a Microbial Fuel Cell (MFC) and shown to increase the 1,3‐PDO yield from 0.57 to 0.89 mol_1,3‐PDO_ mol^−1^_glycerol_ under anodic conditions compared to conventional fermentation (Narcizo et al. 2023). This strain was part of an anodic community primarily composed of *Citrobacter* and *Klebsiella* species (both recognised as 1,3‐PDO producers), and it was initially thought to act solely as an electron shuttle supplier. The observation of 1,3‐PDO production by *P. aeruginosa* is noteworthy, since this species usually relies on the Entner–Doudoroff pathway rather than the Embden–Meyerhof route commonly associated with fermentative PDO synthesis. However, genome analysis revealed the presence of three copies of the *dhaT* gene, which encodes 1,3‐propanediol dehydrogenase, and the electro‐fermentative conditions likely facilitated this atypical metabolic outcome. Electrochemical characterisation suggests that electron transfer to the anode occurs both directly and indirectly and that the microbial biofilm serves as an electroactive interface facilitating this transfer (Narcizo et al. 2023, 2025).

If this behaviour is generalised to other species, selecting mutants or microbial consortia capable of growing at low extracellular ORP or utilising cathodic electrons could be an efficient strategy for enhancing 1,3‐PDO production from glycerol. However, this approach requires further studies for industrial‐scale applications due to the diversity of electron transfer mechanisms and the specific responses to particular potential or ORP conditions, which are highly strain dependent.

## 5 Conclusions and Outlooks

Organic waste valorisation by fermentation for producing commodity chemicals has been the focus of a growing number of environmental policies and research projects. Although glycerol from the biodiesel industry has intrinsic value and cannot be strictly considered as waste, it requires costly purification steps to be used in the pharmaceutical, cosmetic or food industries. Biological processes offer the possibility to upgrade crude glycerol into 1,3‐PDO or other value‐added commodity chemicals after only a few upstream purification steps that are already carried out in most biodiesel production plants (i.e., salts and methanol recycling). While the composition of crude glycerol can vary significantly between different biodiesel production processes, it is generally consistent within a given process, which can facilitate the implementation of biological conversions.

However, the opportunity for an environmentally friendly crude glycerol‐based 1,3‐PDO production is not fully seized by the main industrial actors. Indeed, part of the 1,3‐PDO production is still from glucose, whose utilisation for the production of chemical commodities competes directly with food production. One of the main reasons is that the glycerol market is still highly volatile as it largely depends on biofuel policies (Anitha et al. 2016; Attarbachi et al. 2023) and its utilisation as substrate presents a risk for the 1,3‐PDO‐producing companies. Nonetheless, several glycerol‐based processes have been developed at industrial scale, using genetically modified strains to reach high 1,3‐PDO concentrations. Although this feature is highly desirable to decrease downstream process costs, the use of genetically modified strains also involves several drawbacks such as the requirement for refined glycerol as substrate and sterile conditions that drastically increase the operational costs. The possible alternatives include wild‐type strains or mixed microbial cultures (see Table 3) resistant to crude glycerol impurities and capable of tolerating a range of compositions, facilitating their use across different biodiesel production streams. Open mixed cultures have recently raised an increasing interest since they can ferment glycerol under non‐sterile conditions and without expensive vitamin addition while offering significant performances for 1,3‐PDO production. Optimising population selection procedures in mixed‐culture glycerol fermentation could help structure efficient microbial consortia that could be more competitive in terms of operating costs with genetically modified organism‐based fermentations. Nevertheless, techno‐economic studies should be carried out to assess if these lower fermentation operating costs could mitigate the increase of downstream costs related to the lower 1,3‐PDO concentrations attained so far by these processes.

Apart from the choice of an appropriate inoculum, there is still room for further optimisation of 1,3‐PDO production through appropriate process configuration and parameters. Process engineering of glycerol fermentation has been extensively explored over the past 20 years and has conducted industrial processes to carry out fed‐batch systems. Indeed, this configuration is the most efficient for reaching high 1,3‐PDO concentrations while avoiding inhibitory effects related to glycerol at high concentrations. While continuous systems display significantly higher productivities, the highest 1,3‐PDO concentrations attained so far with this approach are still not compatible with the existing downstream processes. Further improvement could be related to cell immobilisation or recirculation strategies that seem promising for future process intensification, both for fed‐batch and continuous systems. Another interesting alternative that has been recently proposed is the use of bio‐electrochemical reactors (i.e., electro‐fermentation systems). Indeed, the two environmental parameters identified as having the most influence on glycerol fermentation are pH and ORP. If pH control can be easily implemented, ORP regulation can be much more difficult and expensive to be carried out. Nevertheless, this parameter seems particularly relevant as 1,3‐PDO production is directly related to bacterial intracellular redox state. So far, the use of electrodes poised at a defined redox potential has shown to have a strong influence on both glycerol fermentation patterns and kinetics. Nonetheless, most experiments carried out until now have only shown macroscopic effects of electro‐fermentation while only little is known about the underlying mechanisms. More fundamental research on this matter could unveil new levers that could be key elements for future efficient glycerol fermentation processes for the production of 1,3‐PDO.

## Author Contributions

**María Fernanda Pérez‐Bernal:** investigation, writing – review and editing, visualization. **Roman Moscoviz:** writing – original draft, writing – review and editing, investigation, visualization, conceptualization. **Xiaoli Wang:** investigation, writing – review and editing. **Nicolas Bernet:** supervision, resources, project administration, funding acquisition, investigation, writing – review and editing. **Eric Trably:** supervision, project administration, investigation, writing – reviewing and editing.

## Conflicts of Interest

The authors declare no conflicts of interest.

## Data Availability

Data sharing not applicable to this article as no data sets were generated or analysed during the current study.

## References

[mbt270265-bib-0001] Akram, F. , I. u. Haq , S. I. Raja , et al. 2022. “Current Trends in Biodiesel Production Technologies and Future Progressions: A Possible Displacement of the Petro‐Diesel.” Journal of Cleaner Production 370: 133479.

[mbt270265-bib-0002] Almeida, J. R. M. , L. C. L. Fávaro , and B. F. Quirino . 2012. “Biodiesel Biorefinery: Opportunities and Challenges for Microbial Production of Fuels and Chemicals From Glycerol Waste.” Biotechnology for Biofuels 5: 48. 22809320 10.1186/1754-6834-5-48 PMC3467170

[mbt270265-bib-0003] Anitha, M. , S. K. Kamarudin , and N. T. Kofli . 2016. “The Potential of Glycerol as a Value‐Added Commodity.” Chemical Engineering Journal 295: 119–130.

[mbt270265-bib-0004] Atsumi, S. , A. F. Cann , M. R. Connor , et al. 2008. “Metabolic Engineering of *Escherichia coli* for 1‐Butanol Production.” Metabolic Engineering 10: 305–311. 17942358 10.1016/j.ymben.2007.08.003

[mbt270265-bib-0005] Attarbachi, T. , M. D. Kingsley , and V. Spallina . 2023. “New Trends on Crude Glycerol Purification: A Review.” Fuel 340: 127485.

[mbt270265-bib-0006] Avci, F. G. , D. Huccetogullari , and N. Azbar . 2014. “The Effects of Cell Recycling on the Production of 1,3‐Propanediol by *Klebsiella pneumoniae* .” Bioprocess and Biosystems Engineering 37: 513–519. 23892658 10.1007/s00449-013-1018-z

[mbt270265-bib-0007] Ayoub, M. , and A. Z. Abdullah . 2012. “Critical Review on the Current Scenario and Significance of Crude Glycerol Resulting From Biodiesel Industry Towards More Sustainable Renewable Energy Industry.” Renewable and Sustainable Energy Reviews 16: 2671–2686.

[mbt270265-bib-0008] Barbirato, F. , C. Camarasa‐Claret , J. P. Grivet , and A. Bories . 1995. “Glycerol Fermentation by a New 1,3‐Propanediol‐Producing Microorganism: *Enterobacter agglomerans* .” Applied Microbiology and Biotechnology 43: 786–793.

[mbt270265-bib-0009] Barbirato, F. , E. H. Himmi , T. Conte , and A. Bories . 1998. “1,3‐Propanediol Production by Fermentation: An Interesting Way to Valorize Glycerin From the Ester and Ethanol Industries.” Industrial Crops and Products 7: 281–289.

[mbt270265-bib-0010] Bardhan, S. K. , S. Gupta , M. E. Gorman , and M. A. Haider . 2015. “Biorenewable Chemicals: Feedstocks, Technologies and the Conflict With Food Production.” Renewable and Sustainable Energy Reviews 51: 506–520.

[mbt270265-bib-0011] Berthomieu, R. , M. F. Pérez‐Bernal , G. Santa‐Catalina , E. Desmond‐Le Quéméner , N. Bernet , and E. Trably . 2022. “Mechanisms Underlying *Clostridium pasteurianum* 's Metabolic Shift When Grown With *Geobacter sulfurreducens* .” Applied Microbiology and Biotechnology 106: 865–876. 34939136 10.1007/s00253-021-11736-7

[mbt270265-bib-0012] Bharathiraja, B. , T. Sudharsanaa , A. Bharghavi , J. Jayamuthunagai , and R. Praveenkumar . 2016. “Biohydrogen and Biogas—An Overview on Feedstocks and Enhancement Process.” Fuel 185: 810–828.

[mbt270265-bib-0014] Biebl, H. 1991. “Glycerol Fermentation of 1,3‐Propanediol by *Clostridium butyricum* . Measurement of Product Inhibition by Use of a pH‐Auxostat.” Applied Microbiology and Biotechnology 35: 701–705.

[mbt270265-bib-0013] Biebl, H. 2001. “Fermentation of Glycerol by *Clostridium pasteurianum* —Batch and Continuous Culture Studies.” Journal of Industrial Microbiology & Biotechnology 27: 18–26. 11598806 10.1038/sj.jim.7000155

[mbt270265-bib-0015] Biebl, H. , K. Menzel , A.‐P. Zeng , and W.‐D. Deckwer . 1999. “Microbial Production of 1,3‐Propanediol.” Applied Microbiology and Biotechnology 52: 289–297. 10531640 10.1007/s002530051523

[mbt270265-bib-0016] Bizukojc, M. , D. Dietz , J. Sun , and A.‐P. Zeng . 2010. “Metabolic Modelling of Syntrophic‐Like Growth of a 1,3‐Propanediol Producer, *Clostridium butyricum*, and a Methanogenic Archeon, *Methanosarcina mazei* , Under Anaerobic Conditions.” Bioprocess and Biosystems Engineering 33: 507–523. 19680695 10.1007/s00449-009-0359-0

[mbt270265-bib-0017] Bode, H. B. 2006. “No Need to be Pure: Mix the Cultures!” Chemistry & Biology 13: 1245–1246. 17185219 10.1016/j.chembiol.2006.12.001

[mbt270265-bib-0018] Boenigk, R. , S. Bowien , and G. Gottschalk . 1993. “Fermentation of Glycerol to 1,3‐Propanediol in Continuous Cultures of *Citrobacter freundii* .” Applied Microbiology and Biotechnology 38: 453–457.

[mbt270265-bib-0019] Chen, X. , Z. Xiu , J. Wang , D. Zhang , and P. Xu . 2003. “Stoichiometric Analysis and Experimental Investigation of Glycerol Bioconversion to 1,3‐Propanediol by *Klebsiella pneumoniae* Under Microaerobic Conditions.” Enzyme and Microbial Technology 33: 386–394.

[mbt270265-bib-0020] Chen, Y. , J. J. Cheng , and K. S. Creamer . 2008. “Inhibition of Anaerobic Digestion Process: A Review.” Bioresource Technology 99: 4044–4064. 17399981 10.1016/j.biortech.2007.01.057

[mbt270265-bib-0021] Cheng, K.‐K. , H.‐J. Liu , and D.‐H. Liu . 2005. “Multiple Growth Inhibition of *Klebsiella pneumoniae* in 1,3‐Propanediol Fermentation.” Biotechnology Letters 27: 19–22. 15685414 10.1007/s10529-004-6308-8

[mbt270265-bib-0022] Choi, O. , T. Kim , H. M. Woo , and Y. Um . 2014. “Electricity‐Driven Metabolic Shift Through Direct Electron Uptake by Electroactive Heterotroph *Clostridium pasteurianum* .” Scientific Reports 4: 6961. 25376371 10.1038/srep06961 PMC4223642

[mbt270265-bib-0023] Ciriminna, R. , C. D. Pina , M. Rossi , and M. Pagliaro . 2014. “Understanding the Glycerol Market.” European Journal of Lipid Science and Technology 116: 1432–1439.

[mbt270265-bib-0024] Clomburg, J. M. , and R. Gonzalez . 2013. “Anaerobic Fermentation of Glycerol: A Platform for Renewable Fuels and Chemicals.” Trends in Biotechnology 31: 20–28. 23178075 10.1016/j.tibtech.2012.10.006

[mbt270265-bib-0025] Coherent Market Insights . 2025. Global 1,3 Propanediol Market Size & Analysis, 2025–2032. Coherent Mark Insights.

[mbt270265-bib-0026] Colin, T. , A. Bories , C. Lavigne , and G. Moulin . 2001. “Effects of Acetate and Butyrate During Glycerol Fermentation by *Clostridium butyricum* .” Current Microbiology 43: 238–243. 11683356 10.1007/s002840010294

[mbt270265-bib-0027] Colin, T. , A. Bories , and G. Moulin . 2000. “Inhibition of *Clostridium butyricum* by 1,3‐Propanediol and Diols During Glycerol Fermentation.” Applied Microbiology and Biotechnology 54: 201–205. 10968633 10.1007/s002530000365

[mbt270265-bib-0028] Crespo, C. , T. Pozzo , E. Nordberg Karlsson , M. T. Alvarez , and B. Mattiasson . 2012. “ *Caloramator boliviensis* sp. nov., a Thermophilic, Ethanol‐Producing Bacterium Isolated From a Hot Spring.” International Journal of Systematic and Evolutionary Microbiology 62: 1679–1686. 21908677 10.1099/ijs.0.032664-0

[mbt270265-bib-0029] da Silva, G. P. , M. Mack , and J. Contiero . 2009. “Glycerol: A Promising and Abundant Carbon Source for Industrial Microbiology.” Biotechnology Advances 27: 30–39. 18775486 10.1016/j.biotechadv.2008.07.006

[mbt270265-bib-0030] da Silva Ruy, A. D. , R. M. de Brito Alves , T. L. Reis Hewer , D. de Aguiar Pontes , L. S. Gomes Teixeira , and L. A. Magalhães Pontes . 2021. “Catalysts for Glycerol Hydrogenolysis to 1,3‐Propanediol: A Review of Chemical Routes and Market.” Catalysis Today 381: 243–253.

[mbt270265-bib-0031] Dabrock, B. , H. Bahl , and G. Gottschalk . 1992. “Parameters Affecting Solvent Production by *Clostridium pasteurianum* .” Applied and Environmental Microbiology 58: 1233–1239. 16348691 10.1128/aem.58.4.1233-1239.1992 PMC195580

[mbt270265-bib-0032] Dahiya, S. , and V. S. Mohan . 2021. “Selective Enrichment of Mixed Consortia Towards Enhanced 1,3‐Propanediol Production From Glycerol.” Sustainable Energy Technologies and Assessments 47: 101337.

[mbt270265-bib-0033] Dietz, D. , and A.‐P. Zeng . 2014. “Efficient Production of 1,3‐Propanediol From Fermentation of Crude Glycerol With Mixed Cultures in a Simple Medium.” Bioprocess and Biosystems Engineering 37: 225–233. 23749235 10.1007/s00449-013-0989-0

[mbt270265-bib-0034] Drozdzynska, A. , K. Leja , and K. Czaczyk . 2011. “Biotechnological Production of 1,3‐Propanediol From Crude Glycerol.” BioTechnologia: Journal of Biotechnology, Computational Biology and Bionanotechnology 92: 92–100.

[mbt270265-bib-0035] Drozdzynska, A. , J. Pawlicka , P. Kubiak , et al. 2014. “Conversion of Glycerol to 1,3‐Propanediol by *Citrobacter freundii* and *Hafnia alvei* —Newly Isolated Strains From the *Enterobacteriaceae* .” New Biotechnology 31: 402–410. 24768868 10.1016/j.nbt.2014.04.002

[mbt270265-bib-0036] Du, C. , H. Yan , Y. Zhang , Y. Li , and Z. Cao . 2006. “Use of Oxidoreduction Potential as an Indicator to Regulate 1,3‐Propanediol Fermentation by *Klebsiella pneumoniae* .” Applied Microbiology and Biotechnology 69: 554–563. 16021488 10.1007/s00253-005-0001-2

[mbt270265-bib-0037] Du, C. , Y. Zhang , Y. Li , and Z. Cao . 2007. “Novel Redox Potential‐Based Screening Strategy for Rapid Isolation of *Klebsiella pneumoniae* Mutants With Enhanced 1,3‐Propanediol‐Producing Capability.” Applied and Environmental Microbiology 73: 4515–4521. 17513581 10.1128/AEM.02857-06 PMC1932824

[mbt270265-bib-0038] E4tech, RE‐CORD & WUR . 2015. “From the Sugar Platform to Biofuels and Biochemicals.” Final report for the European Commission, Contract No EN ER/C2/423‐2012/SI2.673791. London: E4tech (UK) Ltd. Available at: https://ibcarb.com/wp‐content/uploads/EC‐Sugar‐Platform‐final‐report.pdf.

[mbt270265-bib-0039] Erickson, B. , J. E. Nelson , and P. Winters . 2012. “Perspective on Opportunities in Industrial Biotechnology in Renewable Chemicals.” Biotechnology Journal 7: 176–185. 21932250 10.1002/biot.201100069 PMC3490365

[mbt270265-bib-0040] Fokum, E. , H. M. Zabed , Q. Guo , et al. 2019. “Metabolic Engineering of Bacterial Strains Using CRISPR/Cas9 Systems for Biosynthesis of Value‐Added Products.” Food Bioscience 28: 125–132.

[mbt270265-bib-0041] Fokum, E. , H. M. Zabed , J. Yun , G. Zhang , and X. Qi . 2021. “Recent Technological and Strategical Developments in the Biomanufacturing of 1,3‐Propanediol From Glycerol.” International journal of Environmental Science and Technology 18: 2467–2490.

[mbt270265-bib-0042] Gallardo, R. , C. Faria , L. R. Rodrigues , M. A. Pereira , and M. M. Alves . 2014. “Anaerobic Granular Sludge as a Biocatalyst for 1,3‐Propanediol Production From Glycerol in Continuous Bioreactors.” Bioresource Technology 155: 28–33. 24413479 10.1016/j.biortech.2013.12.008

[mbt270265-bib-0043] Ghosh, S. , R. Chowdhury , and P. Bhattacharya . 2016. “Mixed Consortia in Bioprocesses: Role of Microbial Interactions.” Applied Microbiology and Biotechnology 100: 4283–4295. 27037693 10.1007/s00253-016-7448-1

[mbt270265-bib-0044] González‐Pajuelo, M. , J. C. Andrade , and I. Vasconcelos . 2005. “Production of 1,3‐Propanediol by *Clostridium butyricum* VPI 3266 in Continuous Cultures With High Yield and Productivity.” Journal of Industrial Microbiology & Biotechnology 32: 391–396. 16044292 10.1007/s10295-005-0012-0

[mbt270265-bib-0045] González‐Pajuelo, M. , I. Meynial‐Salles , F. Mendes , J. C. Andrade , I. Vasconcelos , and P. Soucaille . 2005. “Metabolic Engineering of *Clostridium acetobutylicum* for the Industrial Production of 1,3‐Propanediol From Glycerol.” Metabolic Engineering 7: 329–336. 16095939 10.1016/j.ymben.2005.06.001

[mbt270265-bib-0046] Gottschal, J. C. , A. C. Alderkamp , S. J. H. W. O. Elferink , et al. 2002. “ *Lactobacillus diolivorans* sp. nov., a 1,2‐Propanediol‐Degrading Bacterium Isolated From Aerobically Stable Maize Silage.” International Journal of Systematic and Evolutionary Microbiology 52: 639–646. 11931178 10.1099/00207713-52-2-639

[mbt270265-bib-0047] Grahame, D. A. S. , T. S. Kang , N. H. Khan , and T. Tanaka . 2013. “Alkaline Conditions Stimulate the Production of 1,3‐Propanediol in *Lactobacillus pains* PM1 Through Shifting Metabolic Pathways.” World Journal of Microbiology and Biotechnology 29: 1207–1215. 23400350 10.1007/s11274-013-1283-7

[mbt270265-bib-0048] Gungormusler, M. , C. Gonen , and N. Azbar . 2011a. “Continuous Production of 1,3‐Propanediol Using Raw Glycerol With Immobilized *Clostridium beijerinckii* NRRL B‐593 in Comparison to Suspended Culture.” Bioprocess and Biosystems Engineering 34: 727–733. 21336641 10.1007/s00449-011-0522-2

[mbt270265-bib-0049] Gungormusler, M. , C. Gonen , and N. Azbar . 2011b. “Use of Ceramic‐Based Cell Immobilization to Produce 1,3‐Propanediol From Biodiesel‐Derived Waste Glycerol With *Klebsiella pneumoniae*: PDO Production via Immobilization.” Journal of Applied Microbiology 111: 1138–1147. 21883732 10.1111/j.1365-2672.2011.05137.x

[mbt270265-bib-0050] Gungormusler‐Yilmaz, M. , N. Cicek , D. B. Levin , and N. Azbar . 2015. “Cell Immobilization for Microbial Production of 1,3‐Propanediol.” Critical Reviews in Biotechnology 36, no. 3: 482–494. 25600463 10.3109/07388551.2014.992386

[mbt270265-bib-0051] Guo, Y. , L. Dai , B. Xin , et al. 2017. “1,3‐Propanediol Production by a Newly Isolated Strain, *Clostridium perfringens* GYL.” Bioresource Technology 233: 406–412. 28315821 10.1016/j.biortech.2017.02.116

[mbt270265-bib-0052] Hallenbeck, P. C. , M. Abo‐Hashesh , and D. Ghosh . 2012. “Strategies for Improving Biological Hydrogen Production.” Bioresource Technology 110: 1–9. 22342581 10.1016/j.biortech.2012.01.103

[mbt270265-bib-0053] Hallenbeck, P. C. 2013. “Chapter 2 ‐ Fundamentals of Biohydrogen A2—Pandey, Ashok.” In Biohydrogen, edited by J.‐S. Chang , P. C. Hallenbecka , and C. Larroche , 25–43. Elsevier.

[mbt270265-bib-0054] Harrington, T. D. , V. N. Tran , A. Mohamed , et al. 2015. “The Mechanism of Neutral Red‐Mediated Microbial Electrosynthesis in *Escherichia coli* : Menaquinone Reduction.” Bioresource Technology 192: 689–695. 26094195 10.1016/j.biortech.2015.06.037 PMC4516386

[mbt270265-bib-0055] Hiremath, A. , M. Kannabiran , and V. Rangaswamy . 2011. “1,3‐Propanediol Production From Crude Glycerol From Jatropha Biodiesel Process.” New Biotechnology 28: 19–23. 20601262 10.1016/j.nbt.2010.06.006

[mbt270265-bib-0056] Homann, T. , C. Tag , H. Biebl , W.‐D. Deckwer , and B. Schink . 1990. “Fermentation of Glycerol to 1,3‐Propanediol by *Klebsiella* and *Citrobacter* Strains.” Applied Microbiology and Biotechnology 33: 121–126. 10.1007/s002530051251 9720196

[mbt270265-bib-0057] Huffer, S. , M. E. Clark , J. C. Ning , H. W. Blanch , and D. S. Clark . 2011. “Role of Alcohols in Growth, Lipid Composition, and Membrane Fluidity of Yeasts, Bacteria, and Archaea.” Applied and Environmental Microbiology 77: 6400–6408. 21784917 10.1128/AEM.00694-11 PMC3187150

[mbt270265-bib-0058] Ji, X.‐J. , H. Huang , and P.‐K. Ouyang . 2011. “Microbial 2,3‐Butanediol Production: A State‐of‐the‐Art Review.” Biotechnology Advances 29: 351–364. 21272631 10.1016/j.biotechadv.2011.01.007

[mbt270265-bib-0059] Jiang, L. , H. Liu , Y. Mu , Y. Sun , and Z. Xiu . 2017. “High Tolerance to Glycerol and High Production of 1,3‐Propanediol in Batch Fermentations by Microbial Consortium From Marine Sludge.” Engineering in Life Sciences 17: 635–644. 32624809 10.1002/elsc.201600215 PMC6999268

[mbt270265-bib-0060] Jin, C. , M. Yao , H. Liu , C. F. Lee , and J. Ji . 2011. “Progress in the Production and Application of n‐Butanol as a Biofuel.” Renewable and Sustainable Energy Reviews 15: 4080–4106.

[mbt270265-bib-0061] Johnson, E. E. , and L. Rehmann . 2016. “The Role of 1,3‐Propanediol Production in Fermentation of Glycerol by *Clostridium pasteurianum* .” Bioresource Technology 209: 1–7. 26946434 10.1016/j.biortech.2016.02.088

[mbt270265-bib-0062] Jolly, J. , B. Hitzmann , S. Ramalingam , and K. B. Ramachandran . 2014. “Biosynthesis of 1,3‐Propanediol From Glycerol With *Lactobacillus reuteri* : Effect of Operating Variables.” Journal of Bioscience and Bioengineering 118: 188–194. 24525111 10.1016/j.jbiosc.2014.01.003

[mbt270265-bib-0063] Ju, J.‐H. , S.‐Y. Heo , S.‐W. Choi , et al. 2021. “Effective Bioconversion of 1,3‐Propanediol From Biodiesel‐Derived Crude Glycerol Using Organic Acid Resistance–Enhanced *Lactobacillus reuteri* JH83.” Bioresource Technology 337: 125361. 34320778 10.1016/j.biortech.2021.125361

[mbt270265-bib-0064] Jun, S.‐A. , C. Moon , C.‐H. Kang , S. W. Kong , B.‐I. Sang , and Y. Um . 2010. “Microbial Fed‐Batch Production of 1,3‐Propanediol Using Raw Glycerol With Suspended and Immobilized *Klebsiella pneumoniae* .” Applied Biochemistry and Biotechnology 161: 491–501. 19921491 10.1007/s12010-009-8839-x

[mbt270265-bib-0065] Kang, T. S. , D. R. Korber , and T. Tanaka . 2014. “Metabolic Engineering of a Glycerol‐Oxidative Pathway in *Lactobacillus pains* PM1 for Utilization of Bioethanol Thin Stillage: Potential to Produce Platform Chemicals From Glycerol.” Applied and Environmental Microbiology 80: 7631–7639. 25281374 10.1128/AEM.01454-14 PMC4249216

[mbt270265-bib-0066] Kanjilal, B. , I. Noshadi , E. J. Bautista , R. Srivastava , and R. S. Parnas . 2015. “Batch, Design Optimization, and DNA Sequencing Study for Continuous 1,3‐Propanediol Production From Waste Glycerol by a Soil‐Based Inoculum.” Applied Microbiology and Biotechnology 99: 2105–2117. 25480510 10.1007/s00253-014-6259-5

[mbt270265-bib-0067] Kaur, G. , A. K. Srivastava , and S. Chand . 2012. “Advances in Biotechnological Production of 1,3‐Propanediol.” Biochemical Engineering Journal 64: 106–118.

[mbt270265-bib-0068] Kleerebezem, R. , and M. C. van Loosdrecht . 2007. “Mixed Culture Biotechnology for Bioenergy Production.” Current Opinion in Biotechnology 18: 207–212. 17509864 10.1016/j.copbio.2007.05.001

[mbt270265-bib-0069] Kosamia, N. M. , M. Samavi , B. K. Uprety , and S. K. Rakshit . 2020. “Valorization of Biodiesel Byproduct Crude Glycerol for the Production of Bioenergy and Biochemicals.” Catalysts 10: 609.

[mbt270265-bib-0070] Kraus, G. A. 2008. “Synthetic Methods for the Preparation of 1,3‐Propanediol.” CLEAN—Soil, Air, Water 36: 648–651.

[mbt270265-bib-0071] Kubiak, P. , K. Leja , K. Myszka , et al. 2012. “Physiological Predisposition of Various *Clostridium* Species to Synthetize 1,3‐Propanediol From Glycerol.” Process Biochemistry 47: 1308–1319.

[mbt270265-bib-0072] Kumar, L. R. , S. K. Yellapu , R. D. Tyagi , and X. Zhang . 2019. “A Review on Variation in Crude Glycerol Composition, Bio‐Valorization of Crude and Purified Glycerol as Carbon Source for Lipid Production.” Bioresource Technology 293: 122155. 31561979 10.1016/j.biortech.2019.122155

[mbt270265-bib-0073] Lan, Y. , J. Feng , X. Guo , H. Fu , and J. Wang . 2021. “Isolation and Characterization of a Newly Identified *Clostridium butyricum* Strain SCUT343‐4 for 1,3‐Propanediol Production.” Bioprocess and Biosystems Engineering 44: 2375–2385. 34231034 10.1007/s00449-021-02610-x

[mbt270265-bib-0074] Lee, C. S. , M. K. Aroua , W. M. a. W. Daud , et al. 2015. “A Review: Conversion of Bioglycerol Into 1,3‐Propanediol via Biological and Chemical Method.” Renewable and Sustainable Energy Reviews 42: 963–972.

[mbt270265-bib-0075] Lee, J. , T. Islam , S. Cho , N. Arumugam , V. K. Gaur , and S. Park . 2025. “Energy Metabolism Coordination for the Byproduct‐Free Biosynthesis of 1,3‐Propanediol in *Escherichia coli* .” Bioresource Technology 421: 132147. 39923861 10.1016/j.biortech.2025.132147

[mbt270265-bib-0076] Lee, J. H. , M.‐Y. Jung , and M.‐K. Oh . 2018. “High‐Yield Production of 1,3‐Propanediol From Glycerol by Metabolically Engineered *Klebsiella pneumoniae* .” Biotechnology for Biofuels 11: 104. 29657579 10.1186/s13068-018-1100-5 PMC5890353

[mbt270265-bib-0077] Li, C. , K. Lesnik , and H. Liu . 2013. “Microbial Conversion of Waste Glycerol From Biodiesel Production Into Value‐Added Products.” Energies 6: 4739–4768.

[mbt270265-bib-0078] Li, T. , X. Chen , J. Chen , Q. Wu , and G.‐Q. Chen . 2014. “Open and Continuous Fermentation: Products, Conditions and Bioprocess Economy.” Biotechnology Journal 9: 1503–1511. 25476917 10.1002/biot.201400084

[mbt270265-bib-0079] Lindlbauer, K. A. , H. Marx , and M. Sauer . 2017. “Effect of Carbon Pulsing on the Redox Household of *Lactobacillus diolivorans* in Order to Enhance 1,3‐Propanediol Production.” New Biotechnology 34: 32–39. 27769866 10.1016/j.nbt.2016.10.004

[mbt270265-bib-0080] Liu, B. , K. Christiansen , R. Parnas , Z. Xu , and B. Li . 2013. “Optimizing the Production of Hydrogen and 1,3‐Propanediol in Anaerobic Fermentation of Biodiesel Glycerol.” International Journal of Hydrogen Energy 38: 3196–3205.

[mbt270265-bib-0081] Liu, C.‐G. , C. Xue , Y.‐H. Lin , and F.‐W. Bai . 2013. “Redox Potential Control and Applications in Microaerobic and Anaerobic Fermentations.” Biotechnology Advances 31: 257–265. 23178703 10.1016/j.biotechadv.2012.11.005

[mbt270265-bib-0082] Liu, G. , C. Feng , Z. Zhu , Y. Sun , and Z. Xiu . 2023. “Fed‐Batch Self‐Regulated Fermentation of Glucose to co‐Produce Glycerol and 1,3‐Propanediol by Recombinant *Escherichia coli* .” Synthetic Biology Engineering 1: 10008.

[mbt270265-bib-0083] Liu, J. , W. Xu , A. Chistoserdov , and R. K. Bajpai . 2016. “Glycerol Dehydratases: Biochemical Structures, Catalytic Mechanisms, and Industrial Applications in 1,3‐Propanediol Production by Naturally Occurring and Genetically Engineered Bacterial Strains.” Applied Biochemistry and Biotechnology 179: 1073–1100. 27033090 10.1007/s12010-016-2051-6

[mbt270265-bib-0084] Louis, P. , and H. J. Flint . 2009. “Diversity, Metabolism and Microbial Ecology of Butyrate‐Producing Bacteria From the Human Large Intestine.” FEMS Microbiology Letters 294: 1–8. 19222573 10.1111/j.1574-6968.2009.01514.x

[mbt270265-bib-0085] Ma, C. , L. Zhang , J. Dai , and Z. Xiu . 2010. “Relaxing the Coenzyme Specificity of 1,3‐Propanediol Oxidoreductase From *Klebsiella pneumoniae* by Rational Design.” Journal of Biotechnology 146: 173–178. 20156491 10.1016/j.jbiotec.2010.02.005

[mbt270265-bib-0086] Ma, Z. , X. Shentu , Y. Bian , and X. Yu . 2012. “1,3‐Propanediol Production From Glucose by Mixed‐Culture Fermentation of *Zygosacharomyces rouxii* and *Klebsiella pneumonia* .” Engineering in Life Sciences 12: 553–559.

[mbt270265-bib-0087] Marchetti, J. M. , V. U. Miguel , and A. F. Errazu . 2007. “Possible Methods for Biodiesel Production.” Renewable and Sustainable Energy Reviews 11: 1300–1311.

[mbt270265-bib-0088] Marr, A. C. 2024. “1,3‐Propanediol, an Exemplary Bio‐Renewable Organic Platform Chemical.” Advanced Synthesis and Catalysis 366: 4835–4845.

[mbt270265-bib-0089] Mattam, A. J. , J. M. Clomburg , R. Gonzalez , and S. S. Yazdani . 2013. “Fermentation of Glycerol and Production of Valuable Chemical and Biofuel Molecules.” Biotechnology Letters 35: 831–842. 23690047 10.1007/s10529-013-1240-4

[mbt270265-bib-0090] Maxon, W. D. 1955. “Continuous Fermentation.” Applied Microbiology 3: 110–122. 14362484 10.1128/am.3.2.110-122.1955 PMC1057071

[mbt270265-bib-0091] McDowall, J. S. , B. J. Murphy , M. Haumann , T. Palmer , F. A. Armstrong , and F. Sargent . 2014. “Bacterial Formate Hydrogenlyase Complex.” Proceedings of the National Academy of Sciences of the United States of America 111: E3948–E3956. 25157147 10.1073/pnas.1407927111 PMC4183296

[mbt270265-bib-0092] Mei, R. , T. Narihiro , M. K. Nobu , and W.‐T. Liu . 2016. “Effects of Heat Shocks on Microbial Community Structure and Microbial Activity of a Methanogenic Enrichment Degrading Benzoate.” Letters in Applied Microbiology 63: 356–362. 27490172 10.1111/lam.12629

[mbt270265-bib-0093] Menzel, K. , A.‐P. Zeng , and W.‐D. Deckwer . 1997. “High Concentration and Productivity of 1,3‐Propanediol From Continuous Fermentation of Glycerol by *Klebsiella pneumoniae* .” Enzyme and Microbial Technology 20: 82–86.

[mbt270265-bib-0094] Metsoviti, M. , A.‐P. Zeng , A. A. Koutinas , and S. Papanikolaou . 2013. “Enhanced 1,3‐Propanediol Production by a Newly Isolated *Citrobacter freundii* Strain Cultivated on Biodiesel‐Derived Waste Glycerol Through Sterile and Non‐Sterile Bioprocesses.” Journal of Biotechnology 163: 408–418. 23220217 10.1016/j.jbiotec.2012.11.018

[mbt270265-bib-0095] Mittermeier, F. , M. Bäumler , P. Arulrajah , et al. 2023. “Artificial Microbial Consortia for Bioproduction Processes.” Engineering in Life Sciences 23: e2100152. 36619879 10.1002/elsc.202100152 PMC9815086

[mbt270265-bib-0096] Moscoviz, R. , F. de Fouchécour , G. Santa‐Catalina , N. Bernet , and E. Trably . 2017. “Cooperative Growth of *Geobacter sulfurreducens* and *Clostridium pasteurianum* With Subsequent Metabolic Shift in Glycerol Fermentation.” Scientific Reports 7: 44334. 28287150 10.1038/srep44334 PMC5347079

[mbt270265-bib-0097] Moscoviz, R. , J. Toledo‐Alarcón , E. Trably , and N. Bernet . 2016. “Electro‐Fermentation: How to Drive Fermentation Using Electrochemical Systems.” Trends in Biotechnology 34: 856–865. 27178018 10.1016/j.tibtech.2016.04.009

[mbt270265-bib-0098] Moscoviz, R. , E. Trably , and N. Bernet . 2016. “Consistent 1,3‐Propanediol Production From Glycerol in Mixed Culture Fermentation Over a Wide Range of pH.” Biotechnology for Biofuels 9: 32. 26855671 10.1186/s13068-016-0447-8 PMC4744455

[mbt270265-bib-0099] Moscoviz, R. , E. Trably , and N. Bernet . 2017. “Electro‐Fermentation Triggering Population Selection in Mixed‐Culture Glycerol Fermentation.” Microbial Biotechnology 11: 74–83. 28695687 10.1111/1751-7915.12747 PMC5743810

[mbt270265-bib-0100] Murarka, A. , Y. Dharmadi , S. S. Yazdani , and R. Gonzalez . 2008. “Fermentative Utilization of Glycerol by *Escherichia coli* and Its Implications for the Production of Fuels and Chemicals.” Applied and Environmental Microbiology 74: 1124–1135. 18156341 10.1128/AEM.02192-07 PMC2258577

[mbt270265-bib-0101] Nakamura, C. E. , and G. M. Whited . 2003. “Metabolic Engineering for the Microbial Production of 1,3‐Propanediol.” Current Opinion in Biotechnology 14: 454–459. 14580573 10.1016/j.copbio.2003.08.005

[mbt270265-bib-0102] Narcizo, J. P. , M.‐E. Guazzaroni , A. R. de Andrade , and V. Reginatto . 2025. “Unlocking 1,3‐Propanediol Production by *Pseudomonas aeruginosa* Through Electro‐Fermentation.” ACS Omega 10: 45438–45449. 41078824 10.1021/acsomega.5c05374 PMC12509004

[mbt270265-bib-0103] Narcizo, J. P. , L. B. K. Mancilio , M. Pedrino , M.‐E. Guazzaroni , A. R. de Andrade , and V. Reginatto . 2023. “A New *Pseudomonas aeruginosa* Isolate Enhances Its Unusual 1,3‐Propanediol Generation From Glycerol in Bioelectrochemical System.” Catalysts 13: 1133.

[mbt270265-bib-0104] Nimbalkar, P. R. , and M. S. Dharne . 2024. “A Review on Microbial 1, 3‐Propanediol Production: Emerging Strategies, Key Hurdles and Attainable Solutions to Re‐Establish Its Commercial Interest.” Industrial Crops and Products 209: 117961.

[mbt270265-bib-0105] Oh, B.‐R. , J.‐W. Seo , M. H. Choi , and C. H. Kim . 2008. “Optimization of Culture Conditions for 1,3‐Propanediol Production From Crude Glycerol by *Klebsiella pneumoniae* Using Response Surface Methodology.” Biotechnology and Bioprocess Engineering 13: 666–670.

[mbt270265-bib-0106] Oh, B.‐R. , J.‐W. Seo , S.‐Y. Heo , et al. 2012. “Optimization of Culture Conditions for 1,3‐Propanediol Production From Glycerol Using a Mutant Strain of *Klebsiella pneumoniae* .” Applied Biochemistry and Biotechnology 166: 127–137. 22072138 10.1007/s12010-011-9409-6

[mbt270265-bib-0107] Oh, S.‐E. , S. Van Ginkel , and B. E. Logan . 2003. “The Relative Effectiveness of pH Control and Heat Treatment for Enhancing Biohydrogen Gas Production.” Environmental Science & Technology 37: 5186–5190. 14655706 10.1021/es034291y

[mbt270265-bib-0108] Otte, B. , E. Grunwaldt , O. Mahmoud , and S. Jennewein . 2009. “Genome Shuffling in *Clostridium diolis* DSM 15410 for Improved 1,3‐Propanediol Production.” Applied and Environmental Microbiology 75: 7610–7616. 19854917 10.1128/AEM.01774-09 PMC2794092

[mbt270265-bib-0109] Pandey, A. , S. Negi , and C. R. Soccol . 2016. Current Developments in Biotechnology and Bioengineering: Production, Isolation and Purification of Industrial Products. Elsevier.

[mbt270265-bib-0110] Papanikolaou, S. 2000. “High Production of 1,3‐Propanediol From Industrial Glycerol by a Newly Isolated *Clostridium butyricum* Strain.” Journal of Biotechnology 77: 191–208. 10682279 10.1016/s0168-1656(99)00217-5

[mbt270265-bib-0111] Paranhos, A. G. d. O. , and E. L. Silva . 2018. “Optimized 1,3‐Propanediol Production From Crude Glycerol Using Mixed Cultures in Batch and Continuous Reactors.” Bioprocess and Biosystems Engineering 41: 1807–1816. 30167787 10.1007/s00449-018-2003-3

[mbt270265-bib-0112] Pérez‐Bernal, M. F. , R. Berthomieu , E. D.‐L. Quéméner , N. Bernet , and E. Trably . 2024. “Influence of Fumarate on Interspecies Electron Transfer and the Metabolic Shift Induced in *Clostridium pasteurianum* by *Geobacter sulfurreducens* .” Journal of Applied Microbiology 135: lxae122. 38749675 10.1093/jambio/lxae122

[mbt270265-bib-0113] Pflügl, S. , H. Marx , D. Mattanovich , and M. Sauer . 2012. “1,3‐Propanediol Production From Glycerol With *Lactobacillus diolivorans* .” Bioresource Technology 119: 133–140. 22728193 10.1016/j.biortech.2012.05.121

[mbt270265-bib-0114] Pflugmacher, U. , and G. Gottschalk . 1994. “Development of an Immobilized Cell Reactor for the Production of 1,3‐Propanediol by *Citrobacter freundii* .” Applied Microbiology and Biotechnology 41: 313–316.

[mbt270265-bib-0115] Pinyaphong, P. , and A. La‐up . 2024. “Optimization of 1,3‐Propanediol Production From Fermentation of Crude Glycerol by Immobilized *Bacillus pumilus* .” Heliyon 10: e35349. 39170159 10.1016/j.heliyon.2024.e35349 PMC11336579

[mbt270265-bib-0116] Pirt, S. J. 1975. Principles of Microbe and Cell Cultivation. Wiley.

[mbt270265-bib-0117] Reimann, A. , H. Biebl , and W.‐D. Deckwer . 1998. “Production of 1,3‐Propanediol by *Clostridium butyricum* in Continuous Culture With Cell Recycling.” Applied Microbiology and Biotechnology 49: 359–363.

[mbt270265-bib-0118] Research and Markets . 2022. “Biodiesel Global Market to Reach $73.05 Billion by 2030 at a CAGR of 10%.” PR Newswire, 15 July 2022. Available at: https://www.prnewswire.com/news‐releases/biodiesel‐global‐market‐to‐reach‐73‐05‐billion‐by‐2030‐at‐a‐cagr‐of‐10‐301587356.html.

[mbt270265-bib-0119] Rossi, D. M. , E. A. de Souza , S. H. Flôres , and M. A. Z. Ayub . 2013. “Conversion of Residual Glycerol From Biodiesel Synthesis Into 1,3‐Propanediol by a New Strain of *Klebsiella pneumoniae* .” Renewable Energy 55: 404–409.

[mbt270265-bib-0120] Samul, D. , K. Leja , and W. Grajek . 2014. “Impurities of Crude Glycerol and Their Effect on Metabolite Production.” Annales de Microbiologie 64: 891–898. 10.1007/s13213-013-0767-x PMC4119583 25100926

[mbt270265-bib-0121] Saxena, R. K. , P. Anand , S. Saran , and J. Isar . 2009. “Microbial Production of 1,3‐Propanediol: Recent Developments and Emerging Opportunities.” Biotechnology Advances 27: 895–913. 19664701 10.1016/j.biotechadv.2009.07.003

[mbt270265-bib-0122] Schink, B. , D. R. Kremer , and T. A. Hansen . 1987. “Pathway of Propionate Formation From Ethanol in *Pelobacter propionicus* .” Archives of Microbiology 147: 321–327.

[mbt270265-bib-0124] Schütz, H. , and F. Radler . 1984. “Anaerobic Reduction of Glycerol to Propanediol‐1.3 by *Lactobacillus brevis* and *Lactobacillus Buchner* .” Systematic and Applied Microbiology 5: 169–178.

[mbt270265-bib-0125] Selembo, P. A. , J. M. Perez , W. A. Lloyd , and B. E. Logan . 2009. “Enhanced Hydrogen and 1,3‐Propanediol Production From Glycerol by Fermentation Using Mixed Cultures.” Biotechnology and Bioengineering 104: 1098–1106. 19623563 10.1002/bit.22487

[mbt270265-bib-0126] Sen, B. , A. P. Dabir , V. B. Lanjekar , and R. Ranade . 2015. “Isolation and Partial Characterization of a New Strain of *Klebsiella pneumoniae* Capable of High 1,3 Propanediol Production From Glycerol.” Global Journal of Environmental Science and Management 1: 99–108.

[mbt270265-bib-0127] Seyfried, M. , D. Lyon , F. A. Rainey , and J. Wiegel . 2002. “ *Caloramator viterbensis* sp. nov., a Novel Thermophilic, Glycerol‐Fermenting Bacterium Isolated From a Hot Spring in Italy.” International Journal of Systematic and Evolutionary Microbiology 52: 1177–1184. 12148625 10.1099/00207713-52-4-1177

[mbt270265-bib-0128] Sittijunda, S. , and A. Reungsang . 2020. “Valorization of Crude Glycerol Into Hydrogen, 1,3‐Propanediol, and Ethanol in an Up‐Flow Anaerobic Sludge Blanket (UASB) Reactor Under Thermophilic Conditions.” Renewable Energy 161: 361–372.

[mbt270265-bib-0129] Stanbury, P. F. , A. Whitaker , and S. J. Hall . 2016. Principles of Fermentation Technology. Butterworth‐Heinemann.

[mbt270265-bib-0130] Sun, Y. , L. Liang , Y. Zheng , J. Han , and Z. Xiu . 2022. “Improvement of 1,3‐Propanediol Production From Crude Glycerol by Co‐Cultivation of Anaerobic and Facultative Microbes Under Non‐Strictly Anaerobic Conditions.” Biotechnology for Biofuels and Bioproducts 15: 40. 35490247 10.1186/s13068-022-02143-9 PMC9055712

[mbt270265-bib-0131] Szymanowska‐Powałowska, D. 2015. “The Effect of High Concentrations of Glycerol on the Growth, Metabolism and Adaptation Capacity of *Clostridium butyricum* DSP1.” Electronic Journal of Biotechnology 18: 128–133.

[mbt270265-bib-0132] Szymanowska‐Powałowska, D. , and P. Kubiak . 2015. “Effect of 1,3‐Propanediol, Organic Acids, and Ethanol on Growth and Metabolism of *Clostridium butyricum* DSP1.” Applied Microbiology and Biotechnology 99: 3179–3189. 25524700 10.1007/s00253-014-6292-4

[mbt270265-bib-0133] Szymanowska‐Powałowska, D. , J. Piątkowska , and K. Leja . 2013. “Microbial Purification of Postfermentation Medium After 1,3‐PD Production From Raw Glycerol.” BioMed Research International 2013: 949107. 24199204 10.1155/2013/949107 PMC3807725

[mbt270265-bib-0134] Tang, T. , F. Qi , H. Liu , and D. Liu . 2013. “Recent Developments in the Microbial Production of 1,3‐Propanediol.” Biofuels 4: 651–667.

[mbt270265-bib-0135] Tang, X. , Y. Tan , H. Zhu , K. Zhao , and W. Shen . 2009. “Microbial Conversion of Glycerol to 1,3‐Propanediol by an Engineered Strain of *Escherichia coli* .” Applied and Environmental Microbiology 75: 1628–1634. 19139229 10.1128/AEM.02376-08 PMC2655474

[mbt270265-bib-0136] Temudo, M. F. , R. Kleerebezem , and M. van Loosdrecht . 2007. “Influence of the pH on (Open) Mixed Culture Fermentation of Glucose: A Chemostat Study.” Biotechnology and Bioengineering 98: 69–79. 17657773 10.1002/bit.21412

[mbt270265-bib-0137] Temudo, M. F. , G. Muyzer , R. Kleerebezem , and M. C. M. van Loosdrecht . 2008. “Diversity of Microbial Communities in Open Mixed Culture Fermentations: Impact of the pH and Carbon Source.” Applied Microbiology and Biotechnology 80: 1121–1130. 18800185 10.1007/s00253-008-1669-x PMC7419374

[mbt270265-bib-0138] Temudo, M. F. , R. Poldermans , R. Kleerebezem , and M. C. M. van Loosdrecht . 2008. “Glycerol Fermentation by (Open) Mixed Cultures: A Chemostat Study.” Biotechnology and Bioengineering 100: 1088–1098. 18553403 10.1002/bit.21857

[mbt270265-bib-0139] Tholozan, J. L. , J. P. Touzel , E. Samain , J. P. Grivet , G. Prensier , and G. Albagnac . 1992. “ *Clostridium neopropionicum* sp. nov., a Strict Anaerobic Bacterium Fermenting Ethanol to Propionate Through Acrylate Pathway.” Archives of Microbiology 157: 249–257. 1510558 10.1007/BF00245158

[mbt270265-bib-0140] Tobajas, M. , A. F. Mohedano , J. A. Casas , and J. J. Rodríguez . 2009. “Unstructured Kinetic Model for Reuterin and 1,3‐Propanediol Production by *Lactobacillus reuteri* From Glycerol/Glucose Cofermentation.” Journal of Chemical Technology and Biotechnology 84: 675–680.

[mbt270265-bib-0142] Urban, R. A. , and B. R. Bakshi . 2009. “1, 3‐Propanediol From Fossils Versus Biomass: A Life Cycle Evaluation of Emissions and Ecological Resources.” Industrial and Engineering Chemistry Research 48: 8068–8082.

[mbt270265-bib-0141] U.S. Department of Health and Human Services . 2009. Biosafety in Microbiological and Biomedical Laboratories, 5th edn. Washington, DC: U.S. Government Printing Office. Available at: https://www.cdc.gov/labs/BMBL.html.

[mbt270265-bib-0143] Utesch, T. , W. Sabra , C. Prescher , J. Baur , P. Arbter , and A.‐P. Zeng . 2019. “Enhanced Electron Transfer of Different Mediators for Strictly Opposite Shifting of Metabolism in *Clostridium pasteurianum* Grown on Glycerol in a New Electrochemical Bioreactor.” Biotechnology and Bioengineering 116: 1627–1643. 30825383 10.1002/bit.26963

[mbt270265-bib-0144] van Heerden, C. , S. Farzad , and J. F. Görgens . 2023. “Techno‐Economic and Environmental Assessments of 1,3‐Propanediol and Xylooligosaccharide Production Annexed to a Typical Sugar Mill.” ACS Sustainable Chemistry & Engineering 11: 16453–16468.

[mbt270265-bib-0145] Varrone, C. , G. Floriotis , T. M. B. Heggeset , et al. 2017. “Continuous Fermentation and Kinetic Experiments for the Conversion of Crude Glycerol Derived From Second‐Generation Biodiesel Into 1,3 Propanediol and Butyric Acid.” Biochemical Engineering Journal 128: 149–161.

[mbt270265-bib-0146] Vieira, P. B. , B. V. Kilikian , R. V. Bastos , E. A. Perpetuo , and C. A. O. Nascimento . 2015. “Process Strategies for Enhanced Production of 1,3‐Propanediol by *Lactobacillus reuteri* Using Glycerol as a Co‐Substrate.” Biochemical Engineering Journal 94: 30–38.

[mbt270265-bib-0147] Virdis, B. , D. R. Hoelzle , A. Marchetti , et al. 2022. “Electro‐Fermentation: Sustainable Bioproductions Steered by Electricity.” Biotechnology Advances 59: 107950. 35364226 10.1016/j.biotechadv.2022.107950

[mbt270265-bib-0148] Vital, M. , A. C. Howe , and J. M. Tiedje . 2014. “Revealing the Bacterial Butyrate Synthesis Pathways by Analyzing (Meta) Genomic Data.” MBio 5: e00889‐14. 24757212 10.1128/mBio.00889-14 PMC3994512

[mbt270265-bib-0149] Wang, W. , J. Sun , M. Hartlep , W.‐D. Deckwer , and A.‐P. Zeng . 2003. “Combined Use of Proteomic Analysis and Enzyme Activity Assays for Metabolic Pathway Analysis of Glycerol Fermentation by *Klebsiella pneumoniae* .” Biotechnology and Bioengineering 83: 525–536. 12827694 10.1002/bit.10701

[mbt270265-bib-0150] Wang, X.‐L. , J.‐J. Zhou , J.‐T. Shen , Y.‐F. Zheng , Y. Sun , and Z.‐L. Xiu . 2020. “Sequential Fed‐Batch Fermentation of 1,3‐Propanediol From Glycerol by *Clostridium butyricum* DL07.” Applied Microbiology and Biotechnology 104: 9179–9191. 32997204 10.1007/s00253-020-10931-2

[mbt270265-bib-0151] Wang, Y. , F. Tao , J. Ni , C. Li , and P. Xu . 2015. “Production of C3 Platform Chemicals From CO_2_ by Genetically Engineered Cyanobacteria.” Green Chemistry 17: 3100–3110.

[mbt270265-bib-0152] Wang, Y. , Z. Wan , Y. Zhu , et al. 2023. “Enhanced 1,3‐Propanediol Production With High Yield From Glycerol Through a Novel Klebsiella–Shewanella Co‐Culture.” Biotechnology for Biofuels and Bioproducts 16: 50. 36964595 10.1186/s13068-023-02304-4 PMC10039557

[mbt270265-bib-0153] Werpy, T. , and G. Petersen . 2004. Top Value Added Chemicals From Biomass: Volume I—Results of Screening for Potential Candidates From Sugars and Synthesis Gas. Golden, Colorado: National Renewable Energy Laboratory. Available at: https://docs.nrel.gov/docs/fy04osti/35523.pdf.

[mbt270265-bib-0154] Westgate, P. J. , and A. H. Emery . 1990. “Approximation of Continuous Fermentation by Semicontinuous Cultures.” Biotechnology and Bioengineering 35: 437–453. 18592537 10.1002/bit.260350502

[mbt270265-bib-0155] Wilkens, E. , A. K. Ringel , D. Hortig , T. Willke , and K.‐D. Vorlop . 2012. “High‐Level Production of 1,3‐Propanediol From Crude Glycerol by *Clostridium butyricum* AKR102a.” Applied Microbiology and Biotechnology 93: 1057–1063. 21972131 10.1007/s00253-011-3595-6

[mbt270265-bib-0156] Willey, J. M. , L. Sherwood , and C. J. Woolverton . 2008. Prescott, Harley, and Klein's Microbiology. McGraw‐Hill Higher Education.

[mbt270265-bib-0157] Wischral, D. , J. Zhang , C. Cheng , et al. 2016. “Production of 1,3‐Propanediol by *Clostridium beijerinckii* DSM 791 From Crude Glycerol and Corn Steep Liquor: Process Optimization and Metabolic Engineering.” Bioresource Technology 212: 100–110. 27085150 10.1016/j.biortech.2016.04.020

[mbt270265-bib-0158] Wittlich, P. , A. Themann , and K.‐D. Vorlop . 2001. “Conversion of Glycerol to 1,3‐Propanediol by a Newly Isolated Thermophilic Strain.” Biotechnology Letters 23: 463–466.

[mbt270265-bib-0159] Wojtusik, M. , A. Rodríguez , V. Ripoll , V. E. Santos , J. L. García , and F. García‐Ochoa . 2015. “1,3‐Propanediol Production by *Klebsiella oxytoca* NRRL‐B199 From Glycerol. Medium Composition and Operational Conditions.” Biotechnology Reports 6: 100–107. 28626702 10.1016/j.btre.2014.12.010 PMC5466260

[mbt270265-bib-0160] Wong, Y. M. , T. Y. Wu , and J. C. Juan . 2014. “A Review of Sustainable Hydrogen Production Using Seed Sludge via Dark Fermentation.” Renewable and Sustainable Energy Reviews 34: 471–482.

[mbt270265-bib-0161] Xafenias, N. , M. O. Anunobi , and V. Mapelli . 2015. “Electrochemical Startup Increases 1,3‐Propanediol Titers in Mixed‐Culture Glycerol Fermentations.” Process Biochemistry 50: 1499–1508.

[mbt270265-bib-0162] Xue, X. , W. Li , Z. Li , Y. Xia , and Q. Ye . 2010. “Enhanced 1,3‐Propanediol Production by Supply of Organic Acids and Repeated Fed‐Batch Culture.” Journal of Industrial Microbiology & Biotechnology 37: 681–687. 20361229 10.1007/s10295-010-0711-z

[mbt270265-bib-0163] Yamanè, T. , and S. Shimizu . 1984. “Fed‐Batch Techniques in Microbial Processes.” In Bioprocess Parameter Control, 147–194. Springer‐Verlag.

[mbt270265-bib-0164] Yang, G. , J. Tian , and J. Li . 2006. “Fermentation of 1,3‐Propanediol by a Lactate Deficient Mutant of *Klebsiella oxytoca* Under Microaerobic Conditions.” Applied Microbiology and Biotechnology 73: 1017–1024. 16960737 10.1007/s00253-006-0563-7

[mbt270265-bib-0165] Yazdani, S. S. , and R. Gonzalez . 2007. “Anaerobic Fermentation of Glycerol: A Path to Economic Viability for the Biofuels Industry.” Current Opinion in Biotechnology 18: 213–219. 17532205 10.1016/j.copbio.2007.05.002

[mbt270265-bib-0166] Zeng, A.‐P. , and H. Biebl . 2002. “Bulk Chemicals From Biotechnology: The Case of 1,3‐Propanediol Production and the New Trends.” In Tools and Applications of Biochemical Engineering Science, edited by K. Schügerl , A.‐P. Zeng , J. G. Aunins , et al., 239–259. Springer. 10.1007/3-540-45736-4_11 11991182

[mbt270265-bib-0167] Zeng, A.‐P. , H. Biebl , H. Schlieker , and W.‐D. Deckwer . 1993. “Pathway Analysis of Glycerol Fermentation by *Klebsiella pneumoniae* : Regulation of Reducing Equivalent Balance and Product Formation.” Enzyme and Microbial Technology 15: 770–779.

[mbt270265-bib-0168] Zeng, A.‐P. 1996. “Pathway and Kinetic Analysis of 1,3‐Propanediol Production From Glycerol Fermentation by *Clostridium butyricum* .” Bioprocess Engineering 14: 169–175.

[mbt270265-bib-0169] Zeng, A.‐P. , A. Ross , H. Biebl , C. Tag , B. Günzel , and W.‐D. Deckwer . 1994. “Multiple Product Inhibition and Growth Modeling of *Clostridium butyricum* and *Klebsiella pneumoniae* in Glycerol Fermentation: Product Inhibition and Growth Modeling of Glycerol Fermentation.” Biotechnology and Bioengineering 44: 902–911. 18618908 10.1002/bit.260440806

[mbt270265-bib-0170] Zhang, G. , B. Ma , X. Xu , C. Li , and L. Wang . 2007. “Fast Conversion of Glycerol to 1,3‐Propanediol by a New Strain of *Klebsiella pneumoniae* .” Biochemical Engineering Journal 37: 256–260.

[mbt270265-bib-0171] Zhang, S. , N. Merino , A. Okamoto , and P. Gedalanga . 2018. “Interkingdom Microbial Consortia Mechanisms to Guide Biotechnological Applications.” Microbial Biotechnology 11: 833–847. 30014573 10.1111/1751-7915.13300 PMC6116752

[mbt270265-bib-0172] Zhang, Y. , Y. Li , C. Du , M. Liu , and Z. Cao . 2006. “Inactivation of Aldehyde Dehydrogenase: A Key Factor for Engineering 1,3‐Propanediol Production by *Klebsiella pneumoniae* .” Metabolic Engineering 8: 578–586. 16931085 10.1016/j.ymben.2006.05.008

[mbt270265-bib-0173] Zhao, Y.‐N. , G. Chen , and S.‐J. Yao . 2006. “Microbial Production of 1,3‐Propanediol From Glycerol by Encapsulated *Klebsiella pneumoniae* .” Biochemical Engineering Journal 32: 93–99.

[mbt270265-bib-0174] Zhou, J.‐J. , J.‐T. Shen , L.‐L. Jiang , Y.‐Q. Sun , Y. Mu , and Z.‐L. Xiu . 2017. “Selection and Characterization of an Anaerobic Microbial Consortium With High Adaptation to Crude Glycerol for 1,3‐Propanediol Production.” Applied Microbiology and Biotechnology 101: 5985–5996. 28512675 10.1007/s00253-017-8311-8

[mbt270265-bib-0175] Zhou, M. , J. Chen , S. Freguia , K. Rabaey , and J. Keller . 2013. “Carbon and Electron Fluxes During the Electricity Driven 1,3‐Propanediol Biosynthesis From Glycerol.” Environmental Science & Technology 47: 11199–11205. 23947779 10.1021/es402132r

[mbt270265-bib-0176] Zhou, M. , S. Freguia , P. G. Dennis , J. Keller , and K. Rabaey . 2015. “Development of Bioelectrocatalytic Activity Stimulates Mixed‐Culture Reduction of Glycerol in a Bioelectrochemical System: Bioelectrocatalytic Activity in Glycerol‐Fed BESs.” Microbial Biotechnology 8: 483–489. 25817314 10.1111/1751-7915.12240 PMC4408180

[mbt270265-bib-0177] Zhu, C. , and B. Fang . 2013. “Application of a Two‐Stage Temperature Control Strategy to Enhance 1,3‐Propanediol Productivity by *Clostridium butyricum*: Enhancing 1,3‐Propanediol Productivity by *Clostridium butyricum* .” Journal of Chemical Technology and Biotechnology 88: 853–857.

[mbt270265-bib-0178] Zhu, C. , B. Fang , and S. Wang . 2016. “Effects of Culture Conditions on the Kinetic Behavior of 1,3‐Propanediol Fermentation by *Clostridium butyricum* With a Kinetic Model.” Bioresource Technology 212: 130–137. 27089428 10.1016/j.biortech.2016.04.028

[mbt270265-bib-0179] Zhu, Y. , D. Li , G. Bao , et al. 2014. “Metabolic Changes in *Klebsiella oxytoca* in Response to Low Oxidoreduction Potential, as Revealed by Comparative Proteomic Profiling Integrated With Flux Balance Analysis.” Applied and Environmental Microbiology 80: 2833–2841. 24584239 10.1128/AEM.03327-13 PMC3993281

[mbt270265-bib-0180] Zinder, S. H. , T. Anguish , and S. C. Cardwell . 1984. “Selective Inhibition by 2‐Bromoethanesulfonate of Methanogenesis From Acetate in a Thermophilic Anaerobic Digestor.” Applied and Environmental Microbiology 47: 1343–1345. 16346572 10.1128/aem.47.6.1343-1345.1984 PMC240241

[mbt270265-bib-0181] Zong, H. , X. Liu , W. Chen , B. Zhuge , and J. Sun . 2017. “Construction of Glycerol Synthesis Pathway in *Klebsiella pneumoniae* for Bioconversion of Glucose Into 1,3‐Propanediol.” Biotechnology and Bioprocess Engineering 22: 549–555.

